# A chromosome level genome, as well as transcriptomes and metabolomes, insights into genome evolution and the biosynthesis of kaempferol and kaempferol derivatives in *Impatiens balsamina* (Balsaminaceae)

**DOI:** 10.3389/fpls.2026.1725789

**Published:** 2026-02-11

**Authors:** Yunsheng Wang

**Affiliations:** College of Resource and Environment, Baoshan University, Baoshan, Yunnan, China

**Keywords:** genome, *Impatiens*, kaempferol, metabolome, molecular pharmacognosy

## Abstract

*Impatiens balsamina* is a plant with notable medicinal, ornamental, and edible value. However, knowledge of its genome evolution and molecular pharmacognosy remains limited. Here, a multi-omics approaches including genome sequencing, transcriptome and metabolome profiling of roots, leaves, and flowers were integrated performed. A 691.61 Mb chromosome-level draft genome of *I. balsamina* is presented, with annotation revealing 32,949 protein-coding genes. It is proposed that two rounds of whole-genome duplication events may be major drivers of species diversity in Balsaminaceae lineages and *Impatiens*. A considerable number of beneficial secondary metabolites, including dihydrokaempferol, kaempferol, and five kaempferol derivatives (KKDs), accumulated at markedly different levels in the roots, leaves, and flowers of *I. balsamina*. The structural genes *IbCHI*, *IbCHS*, *IbF3H*, and *IbFLS*, as well as the glycosyltransferase IbUGT73C, which are involved in KKD biosynthesis, were identified. In addition, transcription factor genes from the WRKY, bHLH, MYB, and Myb-like families, and a P450 gene were suggested to directly regulate KKD biosynthesis, based on correlation analysis, WGCNA, and protein-protein interactions. Overall, these findings provide insights into the genome evolution and molecular pharmacognosy of *I. balsamina* and offer a foundation for breeding and drug development, not only for *I. balsamina* but also for other Impatiens species.

## 1 Introduction

*Impatiens* L., one of the largest genera among higher plants, has pharmaceutical and horticultural importance (Janssens et al., 2012; Szewczyk, 2018). To date, only a few *Impatiens* genomes have been released, including a contig-level genome draft of *I. balsamina* (https://www.ncbi.nlm.nih.gov/datasets/genome/?taxon=35939; https://ngdc.cncb.ac.cn/gwh/search/advanced/result?search_category=&search_term=&source=0&query_box=%20Impatiens), which greatly limits their utilization and understanding of biology and genetics. *I. balsamina*, a popular species in the *Impatiens* genus, has broad application value (Khare, 2007). It has flowers with unique forms and various colors and is cultivated worldwide as an ornamental plant (Staples and Herbst, 2005; Momtaz et al., 2007; Janssens et al., 2012). It has also been used as a traditional herbal medicine in Asia and exhibits antimicrobial (Yang et al., 2001), antianaphylactic (Yoshimi et al., 2003), anti-inflammatory (Hisae and Kyoko, 2002), antidermatitis (Oku and Ishiguro, 2001), anticancer (Ding et al., 2008), antityrosinase, antioxidant (Koodkaew and Sukonkhajorn, 2019), external wound healing (Hariyanto et al., 2017), antihepatic fibrosis (Hariyanto et al., 2017), and anti-neurodegenerative activities (Kim et al., 2017). I. *balsamina* is also consumed as a vegetable and tea in some regions of China (Li et al., 2017; Pires et al., 2021).

Secondary metabolites (SMs) produced in plant tissues, especially medicinal plants, are important drug resources for humans and animals (Ma et al., 2020; Twaij and Hasan, 2022). Many SMs identified in plants include phenolics, terpenes, alkaloids, and quinones, which have antioxidant, anti-inflammatory, antibacterial, antifungal, antimicrobial, and anticancer effects (Sharma et al., 2022). Healthy SMs usually have lower toxicity and are therefore frequently used as drugs (El-Readi et al., 2021). However, the contents of desirable SMs in plant tissues are often low, and some are produced only in specific “medicinal plants”. Their biosynthesis is also influenced by genetic background or environmental conditions, so extraction from wild plants often does not meet demand (Li et al., 2020; Selwal et al., 2023). Metabolic engineering has been developed as a solution (Birchfield and McIntosh, 2020). To perform metabolic engineering, understanding the molecular basis and genetic mechanisms of SM biosynthesis and regulation is required, and the genes involved and their functions need to be identified (Staniek et al., 2013).

Many SMs, including 48 flavonoids, 14 naphthoquinones, seven coumarins, 81 terpenoids and sterols, and 38 phenols, have been identified in different tissues of *I. balsamina* (Qian et al., 2023). Some of these SMs have high medicinal value. For example, 2-methoxynaphthoquinone has pharmacological activities including antiallergic, antimicrobial, anti-inflammatory, antioxidant, immunomodulatory, antihepatic fibrosis, antitumor, insecticidal, anthelmintic, and enzyme-inhibiting activities (Qian et al., 2023). The molecular basis of 2-methoxynaphthoquinone biosynthesis has been preliminarily investigated (Foong et al., 2020). Kaempferol and kaempferol derivatives (KKDs) have been detected in *I. balsamina* tissues and show antibacterial (Lim et al., 2007) and enzyme-inhibiting activity (Kim et al., 2015a, 2019). KKDs from other plants have diverse medicinal applications, including antifungal, antibacterial, anticancer, anti-inflammatory, antiviral, antioxidant, neuroprotective, and cardioprotective effects (Kashyap et al., 2017; Riaz et al., 2018; Bangar et al., 2023). Some derivatives, such as astragalin, can damage pathological cells without affecting normal cells (Chen and Chen, 2013; Chen et al., 2023a). In many plants, kaempferol is biosynthesized from dihydrokaempferol by flavonol synthase (FLS) and serves as a precursor for quercetin, kaempferid, and other derivatives (Calderón-Montaño et al., 2011). Kaempferol occupies a key upstream node in the flavone and flavonol metabolism network (https://www.kegg.jp/pathway/map00944). Besides FLS, chalcone synthase (CHS), chalcone isomerase (CHI), and naringenin 3-dioxygenase (F3H) also play roles in the biosynthesis of kaempferol and related flavonoids (Alam et al., 2020). The expression of FLS, CHS, CHI, and F3H genes is commonly regulated by transcription factors such as MYB (Du et al., 2024), bZIP (Han et al., 2023), YABBY5 (Kayani et al., 2021), and WRKY (Wang et al., 2018). Kaempferol derivatives, like many flavonoids, are typically glycosylated forms produced by conjugation with sugars such as rutinose, rhamnose, glucose, and galactose through the action of glycosyltransferases (Yonekura-Sakakibara and Saito, 2014; McIntosh and Owens, 2016). The molecular basis and regulatory mechanisms underlying the biosynthesis and accumulation of KKDs, as well as most other beneficial metabolites, in the tissues of *I. balsamina* are still unclear.

In this study, the chromosome-level genome of *I. balsamina*, along with transcriptomes and metabolomes of the roots, leaves, and flowers, were analyzed separately and jointly. The aims were to (1) provide a high-quality reference genome for *Impatiens*, (2) provide comprehensive knowledge of the SM profiles in tissues of *I. balsamina*, and (3) elucidate the molecular basis and genetic mechanism underlying KKD biosynthesis and regulation. The results and corresponding data may contribute to breeding and medical applications of not only *I. balsamina* but also other *Impatiens* species.

## 2 Results

### 2.1 Genome assembly

**Figure 1:**
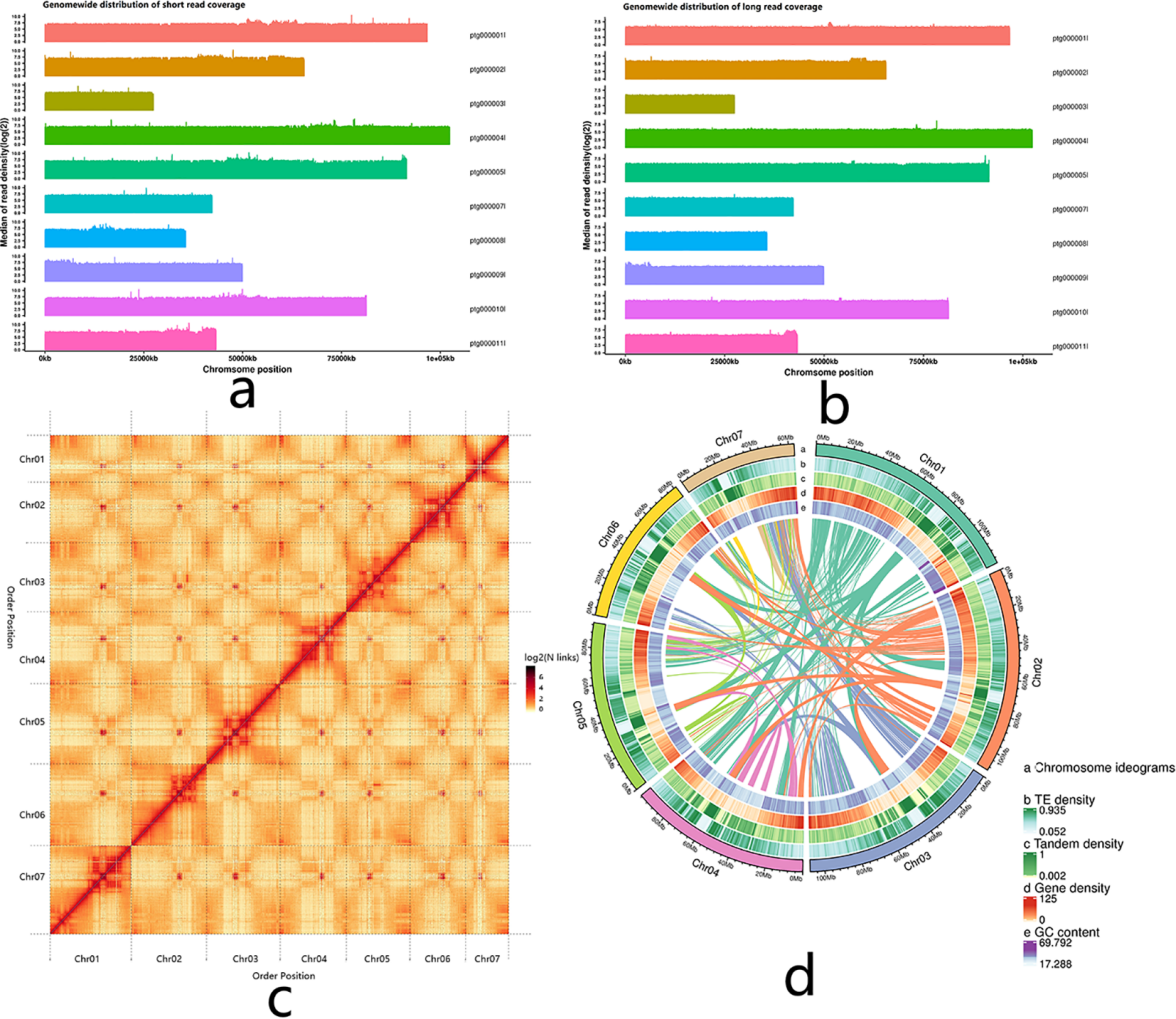
Assembly visualization of the *I*. *balsamina* genome. **(a)** Visualization of short-read data realigned against the assembled contig-level genome. **(b)** Visualization of long-read data realigned against the assembled contig-level genome. **(c)** Visualization of the Hi-C assembly. **(d)** Circular visualization of the seven pseudochromosomes.

First, a 350 bp library of *I. balsamina* was constructed and sequenced, which produced about 53.44 Gb of clean short-read data (**Supplementary Table S1**). K-mer analysis was then performed on these data, and the results indicated that the genome size of the sequenced individuals was about 524.94 Mb, with about 0.45% heterozygosity (**Supplementary Figure S1**). A long-read library of *I. balsamina* was also constructed and sequenced, yielding nearly two million clean CCS totaling 33.25 Gb of data (**Supplementary Table S2**). Based on these data, a primary draft genome (691.608 Mb in length) was assembled, consisting of 405 contigs (N50 = 81.30 Mb) and containing 33.59% GC nucleotides (**Supplementary Table S3**). More than 99% of the sequences of this draft genome could be realigned with the short and long clean sequencing data at 20× coverage, showing that both the sequencing coverage and assembled integrity of the primary contig-level draft genome were high (**Figures 1a, b**; **Supplementary Table S4**).

Finally, a Hi-C library of *I. balsamina* was constructed, sequenced, and produced more than 204 million short reads, with about 53% valid interaction pairs (**Supplementary Table S5**). Based on these valid interaction pairs, the primary contig-level draft genome was further corrected by removing redundancy and assembled into a chromosome-level draft genome, which was 691.609 Mb in length and consisted of 399 scaffolds (N50 = 96.69 Mb) (**Table 1**; **Supplementary Table S6**). Among them, 50 scaffolds (675.23 Mb), containing 13 ordered scaffolds (670.98 Mb), could be mounted on seven chromosomes (**Supplementary Table S7**; **Figure 1c**). These results indicated that most of the genome sequences of *I. balsamina* were successfully assembled into a chromosome-level draft with defined order.

**Table 1:** Overview of the *I. balsamina* genome assembly and annotation.

Items	Counts
Scaffold number	399
Scaffold length	691,609,437 bp
N50 of scaffold	96,691,550 bp
Chromosome mounting ratio	99.37%
Predicting-protein gene number	32,949
Total repetitive sequence length	355,569,899 bp

### 2.2 Genome annotation

A total of 302.57 Mb of retrotransposons dominated by the Copia type (179.49 Mb) and 53.00 Mb of DNA transposons dominated by the CACTA type (7.78 Mb) were identified in the draft genome of *I. balsamina* (**Table 1**; **Supplementary Table S8**). Many tandem repeats containing 192,827 1–9 bp microsatellites were also detected (**Supplementary Table S9**). These are useful molecular tools that can be used for breeding and for research on the genetic and ecological aspects of *I. balsamina* and other *Impatiens* species. Repeat sequences account for about 70% of the *I. balsamina* genome (**Supplementary Tables S8**, **S9**).

Moreover, 32,949 protein-coding genes were predicted from the draft genome (**Table 1**; **Supplementary Table S10**; **Supplementary Figure S2**). Of these, 1,581 are homologs of BUSCO, accounting for 97.96% of 1614 BUSCO genes (**Supplementary Table S11**), indicating that most protein-coding genes in the *I. balsamina* genome have been captured. A total of 30,938 (93.90%) of the predicted protein-coding genes could be annotated by one or more databases (**Supplementary Table S12**; **Dataset 1**). The positions and distribution patterns of repetitive elements and genes are shown in **Figure 1d**. Additionally, diverse non-coding RNA genes, including 11,984 rRNAs, 3,889 tRNAs, 27 miRNAs, 71 snRNAs, and 58 snoRNAs, along with 277 pseudogenes, were identified in the draft genome of *I. balsamina* (**Supplementary Table S13**).

### 2.3 Comparative genomics

To provide insight into the evolutionary history of the *I. balsamina* genome, a comparative genomics analysis was performed using gene datasets containing genes that encode proteins with more than 100 amino acids. These datasets included 12 Magnoliopsida species: *I. balsamina*, *Trifolium pratense*, *Salix purpurea*, *Linum usitatissimum*, *Sesamum indicum*, *Coptis chinensis*, *Magnolia biondii*, *Gossypium arboreum*, *Papaver somniferum*, *Citrus sinensis*, *Litchi chinensis*, and *Ziziphus jujuba*. The genes were clustered into more than 41,235 families (**Supplementary Table S14**; **Supplementary Figure S3**; **Dataset 2**). A total of 28,439 genes of *I. balsamina* were clustered into 16,082 families, and 892 families were species-specific (**Supplementary Table S14**; **Supplementary Figure S4**; **Dataset 2**).

**Figure 2:**
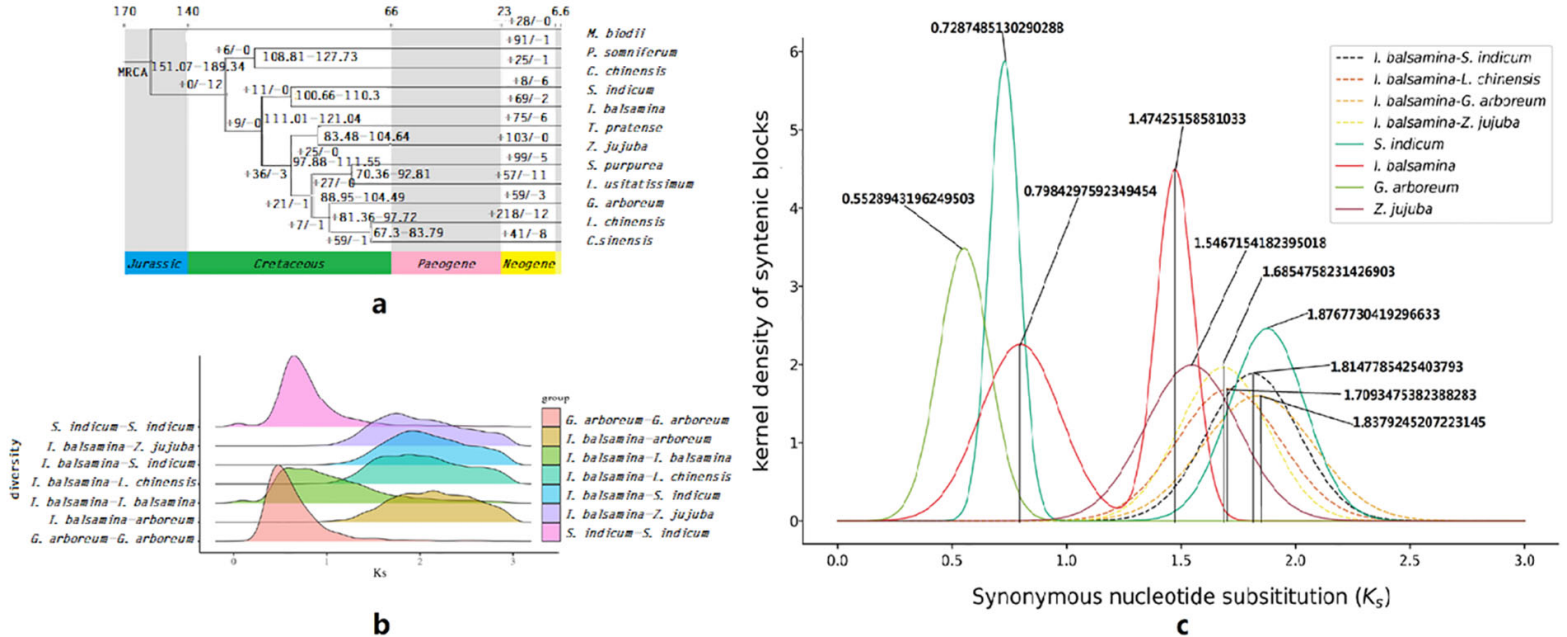
Comparative genomics of *I*. *balsamina.***(a)** Phylogenetic tree of *I*. *balsamina* and the other 11 species. **(b)** Ks distribution of intraspecies homologs and intraspecies paralogs. **(c)** Ks distribution of the kernel density of syntenic blocks.

A phylogenomic tree was constructed using 1,067 single-copy genes encoding protein sequences (**Dataset 3**), the tree topology indicated that *S. indicum* is the closest relative of *I. balsamina* among the 12 compared species and that the *S. indicum* and *I. balsamina* lineages diverged from their common ancestor in the middle Cretaceous period (**Figure 2a**). Significant expansion and contraction were observed in 69 and two gene families of *I. balsamina*, respectively, after the species split from its common ancestor with *S. indicum* ~105.48 million years ago (MYA) (**Figure 2a**). These expanded gene families were enriched mainly in pathways such as “propanoate metabolism” and “fatty acid metabolism” (**Supplementary Figure S5**).

The peak Ks frequency distribution of paralogs suggested that large-scale genome duplication events occurred during the evolution of *I. balsamina* (**Figure 2b**, **Dataset 4**). However, this Ks peak showed a non-normal pattern and had a wide base. This pattern is unlikely to result from a single whole-genome duplication (WGD) event, which typically produces a Ks peak with a normal distribution. Further analysis was performed to estimate the Ks distribution of paralogs in the kernel density of syntenic blocks within or between genomes, and two clear peaks exhibiting a normal distribution were present within the genome of *I. balsamina*, with median Ks values of ~0.8 and ~1.48 (**Figure 2c**).

Moreover, the median Ks of the homologous peak between the genomes of *I. balsamina* and *S. indicum* was ~1.8 (**Figure 2c**, **Dataset 5**). The phylogenetic tree data showed that *I. balsamina* and *S. indicum* diverged from their common ancestor ~105 MYA, corresponding to a Ks value of 1.8. Thus, these two rounds of WGD were inferred to have occurred ~87 MYA and ~47 MYA.

### 2.4 Metabolite profiles of roots, leaves, and flowers

A total of 830 metabolites were identified, including 802, 784, and 744 from the flowers, leaves, and roots of *I. balsamina*, respectively. These included 106 flavonoids, 15 quinones, 91 alkaloids, 71 terpenoids, 99 amino acids, and seven vitamins (**Supplementary Table S15**, **Dataset 6**). The results of the UPLC-MS/MS analysis were confirmed to be reliable by Spearman’s rank correlation analysis and principal component analysis (**Supplementary Figures S6**, **S7**). Among all detected metabolites, 398 (47.95%) could be annotated to 89 metabolic pathways, including 21 (e.g., kaempferol, chalcone, quercetin) in flavonoid biosynthesis and 13 (e.g., kaempferol-3-O-galactoside, myricetin, quercetin-1, astragalin) in flavone and flavonol biosynthesis. Three were in the ubiquinone pathway and other terpenoid-quinone biosynthesis pathways (4-hydroxybenzoic acid, homogentisic acid, L-tyrosine), and 10 were in tropane, piperidine, and pyridine alkaloid biosynthesis pathways (**Supplementary Figure S8**, **Dataset 6**).

The beneficial SMs piperlongumine, 3-hydroxy-2-phenyl-propanamide, 2-methoxynaphthoquinone, asperuloside, and astragalin accumulated at relatively high levels in the flowers, leaves, and roots of *I. balsamina* (**Supplementary Figures S9**-**S11**). Beneficial SMs such as kaempferol-3-O-galactoside, astragalin, kaempferol-3-O-(2’’-O-β-D-glucopyl)-β-D-rutinoside, nicotiflorin, 2’-hydroxygenistein, cyanidin 3-rutinoside, quercetin-1, vasicinol, fulvine, asperuloside, and tulipalin A accumulated in large quantities in flowers (**Supplementary Figure S9**, **Dataset 6**). The accumulation of kuromanin (chloride), cyanidin-3-O-galactoside (chloride), (-)-epicatechin, vasicinol, tombozine, quinones, and R162 was higher in roots (**Supplementary Figure S10**, **Dataset 6**). Kuromanin (chloride), cyanidin-3-O-galactoside (chloride), (-)-epigallocatechin, (-)-epicatechin, cyclo(L-Leu-trans-4-hydroxy-L-Pro), isoammodendrine, vasicinol, tabersonine hydrochloride, N-feruloyloctopamine, quinones (R162), and terpenoids (R)-(+)-citronellal accumulated at high levels in leaves (**Supplementary Figure S11**, **Dataset 6**).

**Figure 3:**
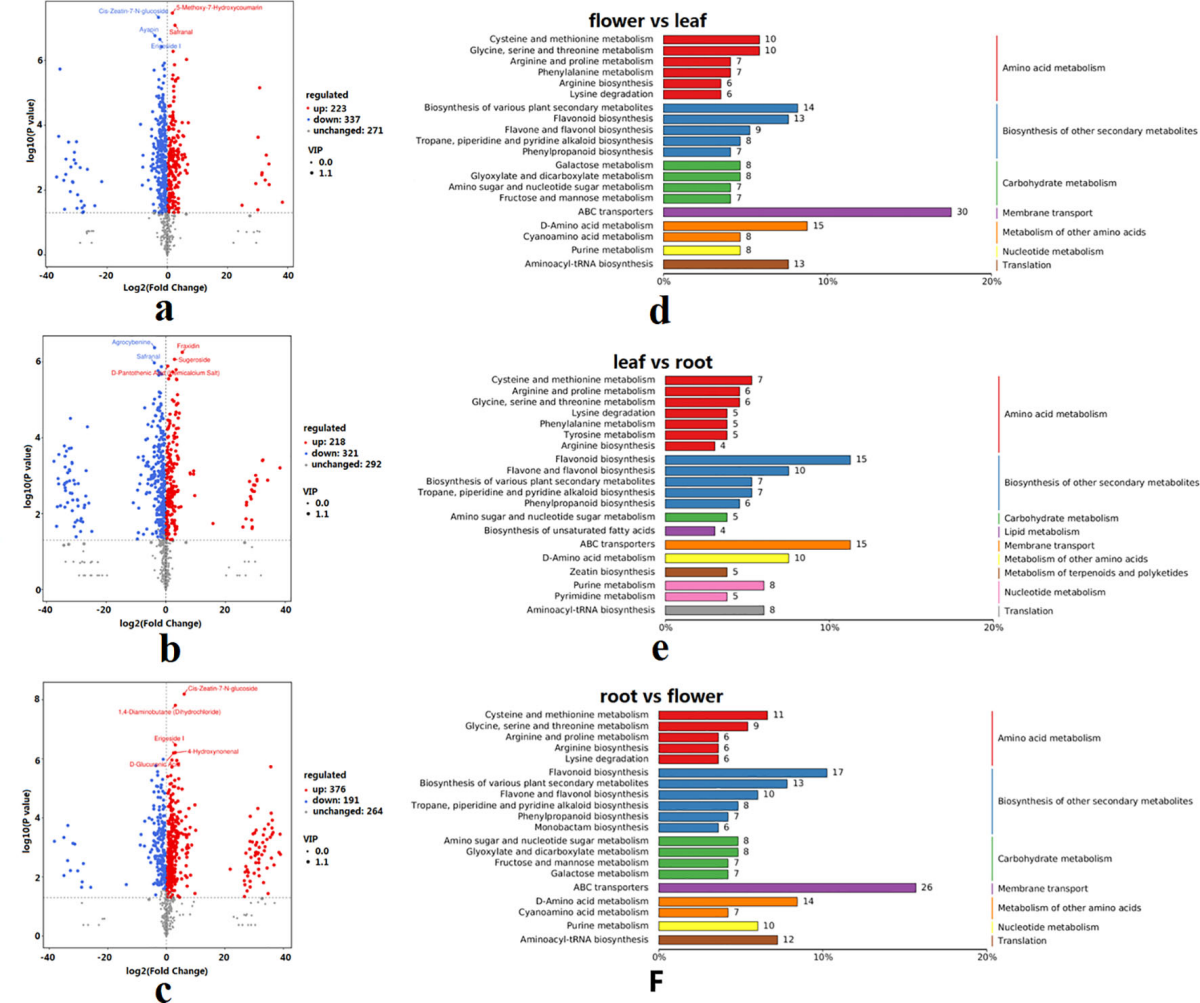
Metabolites in the roots, flowers, and leaves of *I*. *balsamina.***(a)** Differentially accumulated metabolites (DAMs) from flower vs. leaf. **(b)** DAMs from leaf vs. root. **(c)** DAMs from root vs. flower. **(d)** KEGG enrichment of DAMs from flower vs. leaf. **(e)** KEGG enrichment of DAMs from leaf vs. root. **(f)** KEGG enrichment of DAMs from root vs. flower.

Compared to those of leaves, leaves vs. roots, and roots vs. flowers, there were 560, 539, and 567 DAMs, with 223 DAMs (e.g., quercetin-1, kuromanin (chloride), quercetin, kaempferol-3-O-β-glucopyranoside-7-O-α-rhamnopyranoside), 218 DAMs (e.g., arbutin, oleanolic acid, cyanidin-3-O-galactoside (chloride), piperlongumine, lawsone), and 376 DAMs (e.g., arbutin, afzelin, kaempferol, kaempferol-3-O-galactoside, quercetin-1) being upregulated, and 337 DAMs (e.g., afzelin, dihydrokaempferol, kaempferol, kaempferol-3-O-galactoside, astragalin), 321 DAMs (e.g., afzelin, dihydrokaempferol, kaempferol, kaempferol-3-O-galactoside, myricetin), and 191 DAMs (e.g., oleanolic acid, arteminin, cyanidin-3-O-galactoside (chloride), kuromanin (chloride), procyanidin C1) being downregulated, respectively (**Figures 3a–c**; **Dataset 7**). These DAMs, in all three comparisons, were enriched mainly in the metabolic pathways “flavonoid biosynthesis” and “flavone and flavonol biosynthesis” (**Figures 3d–f**).

### 2.5 Transcription profiles of roots, leaves, and flowers

About 20 million short, clean sequencing reads were generated from the transcriptomes of the roots, flowers, and leaves (**Supplementary Table S16**). A total of 82.70–94.99% of these reads could be mapped to the reference genome (**Supplementary Table S17**). Based on the mapping results, 35,373 expressed genes, including 2,424 novel protein-coding genes, were identified, and 3,033 previously predicted protein-coding genes were optimized in structure (**Dataset 7**). A total of 790 new genes were annotated with functions (**Supplementary Table S18**, **Dataset 8**). The gene expression profiles of biologically replicated samples of roots, flowers, and leaves were highly consistent (**Supplementary Figures S12**, **S13**).

Among these expressed genes, 1,988 were predicted to encode transcription factors belonging to 58 families, and 326 genes were predicted to encode different glycosyltransferases (**Dataset 9**). A total of 8,582, 5,701, and 8,391 DEGs were detected in the root vs. flower, flower vs. leaf, and leaf vs. root comparisons, respectively (**Supplementary Figure S14**, **Dataset 10**). The DEGs identified in these comparisons were enriched in plant hormones, signal transduction, the MAPK signaling pathway-plant, phenylpropanoid biosynthesis, and plant-pathogen interactions (**Supplementary Figures S15**-**S17**).

### 2.6 KKD biosynthesis and regulation in roots, leaves, and flowers

**Figure 4:**
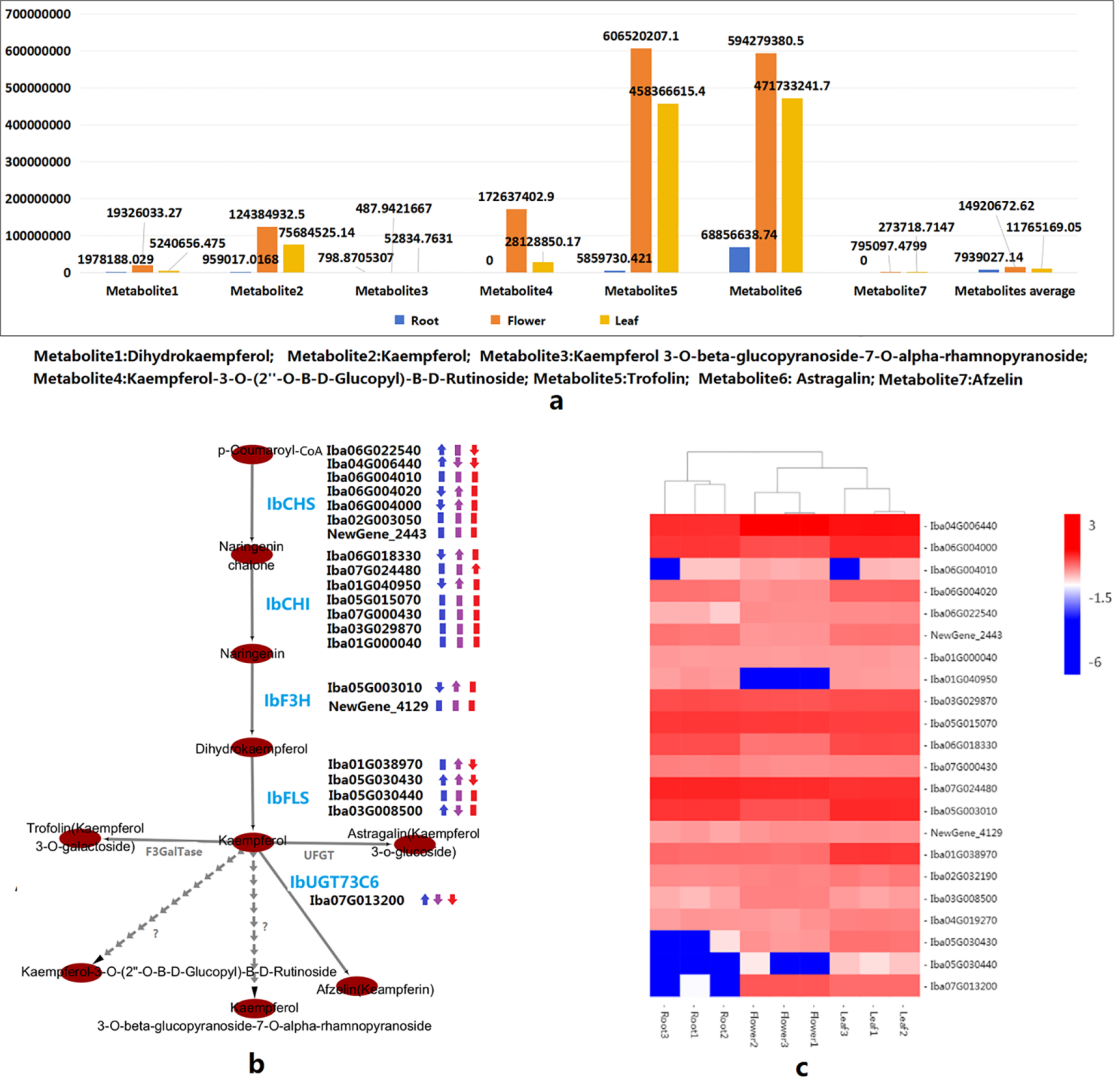
Kaempferol and kaempferol derivatives (KKDs) and their corresponding biosynthetic genes. **(a)** Contents of dihydrokaempferol and KKDs in roots, leaves, and flowers. **(b)** Structural genes responsible for KKD biosynthesis and their expression. **(c)** Heatmap showing the expression levels of structural genes responsible for KKD biosynthesis.

Dihydrokaempferol, kaempferol, and five kaempferol derivatives (kaempferol-3-O-β-glucopyranoside-7-O-α-rhamnopyranoside, kaempferol-3-O-glucoside (astragalin), kaempferol (afzelin), kaempferol-3-O-galactoside (trifolin), and kaempferol-3-O-(2’’-O-β-D-glucopyl)-β-D-rutinoside) were detected in the roots, leaves, and flowers of *I. balsamina.* Except for kaempferol-3-O-β-glucopyranoside-7-O-α-rhamnopyranoside, which showed the highest concentration in leaves and the lowest in flowers, the other six metabolites showed the highest concentration in flowers and the lowest in roots (**Figure 4a**). The concentrations of trifolin and astragalin were relatively high, whereas those of kaempferol-3-O-β-glucopyranoside-7-O-α-rhamnopyranoside were relatively low in the roots, flowers, and leaves of *I. balsamina*; the former two were the top metabolites detected in flowers (**Figure 4a**). High levels of kaempferol accumulated in flowers, whereas kaempferol-3-O-(2’’-O-β-D-glucopyl)-β-D-rutinoside and afzelin were not detected in roots (**Figure 4a**). These results suggest that KKD biosynthesis shows strong organ specificity in *I. balsamina.*

Based on gene annotation in the roots, leaves, and flowers of *I. balsamina*, six *IbCHS* genes, seven *IbCHI* genes, one *IbF3H* gene, and four *IbFLS* genes, which are structural genes potentially responsible for the stepwise biosynthesis of kaempferol from p-coumaroyl, were identified (**Figure 4b**). Among these genes, *Iba04G006440* (*IbCHS*) and *Iba05G015070* (*IbCHI*) were expressed at relatively high levels in leaf, root, and flower tissues (**Figure 4c**). Nine genes were identified as DEGs based on their expression in leaves, roots, and flowers; five DEGs (e.g., *Iba06G004000*, *Iba01G040950*) were downregulated and four DEGs (e.g., *Iba06G022540*, *Iba04G006440*) were upregulated based on roots vs. flowers. Seven DEGs (e.g., *Iba06G004000*, *Iba06G004020*) were upregulated and two DEGs (*Iba04G006440*, *Iba03G008500*) were downregulated based on flowers vs. leaves. One DEG (*Iba07G024480*) was upregulated and four DEGs (e.g., *Iba06G022540*, *Iba04G006440*) were downregulated based on leaves vs. roots (**Figures 4b, c**).

The IbUGT736C coding gene *Iba07G013200* putatively catalyzes the conversion of kaempferol into keampferin, and was upregulated in the root vs. flower comparison but downregulated in the flower vs. leaf and leaf vs. root comparisons (**Figures 4b, c**). The expression levels of the abovementioned DEGs may be the key factors that determine the accumulation of dihydrokaempferol and KKDs in the root, leaf, and flower tissues of *I. balsamina*. Although astragalin and trifolin accumulate at extremely high levels in flowers, the *IbUFGT* gene, encoding flavonol 3-O-glucosyltransferase [2.4.1.91], which putatively catalyzes the conversion of kaempferol into astragalin, and the *IbF3GalTase* gene, encoding kaempferol 3-O-β-D-galactosyltransferase [EC:2.4.1.234], which putatively catalyzes the conversion of kaempferol into trifolin, were not found among the annotated protein-coding genes of the assembled reference genome or annotated expressed genes in the leaves, roots, and flowers *of I. balsamina* (**Figure 4b**). Two possible explanations are that genome sequencing did not capture these two gene sequences, or that other broad-spectrum glycosyltransferases serve the protein functions encoded by specialized *UFGT* or *F3GalTase* genes in *I. balsamina*.

To elucidate the regulatory mechanisms underlying KKD biosynthesis, a correlation analysis between the expression levels of all genes and the accumulation of dihydrokaempferol, kaempferol, kaempferol-3-O-β-glucopyranoside-7-O-α-rhamnopyranoside, astragalin, keampferin (afzelin), kaempferol-3-O-galactoside (trifolin), and kaempferol-3-O-(2’’-O-β-D-glucopyl)-β-D-rutinoside was performed. Many genes, including 13 structural genes, 1,381 TF genes, and 98 glycosyltransferase genes, were related to one or more of these seven metabolites (**Dataset 11**). The intersection pattern of the correlated genes showed that the biosynthesis of kaempferol-3-O-β-glucopyranoside-7-O-α-rhamnopyranoside was regulated by one putative gene set, which differed from another gene set correlated with the six other metabolites. Dihydrokaempferol, kaempferol-3-O-(2’’-O-β-D-glucopyl)-β-D-rutinoside, and keampferin (afzelin) formed one group, whereas kaempferol, trifolin, and astragalin formed another group that shared more commonly correlated genes (**Dataset 11**).

**Figure 5:**
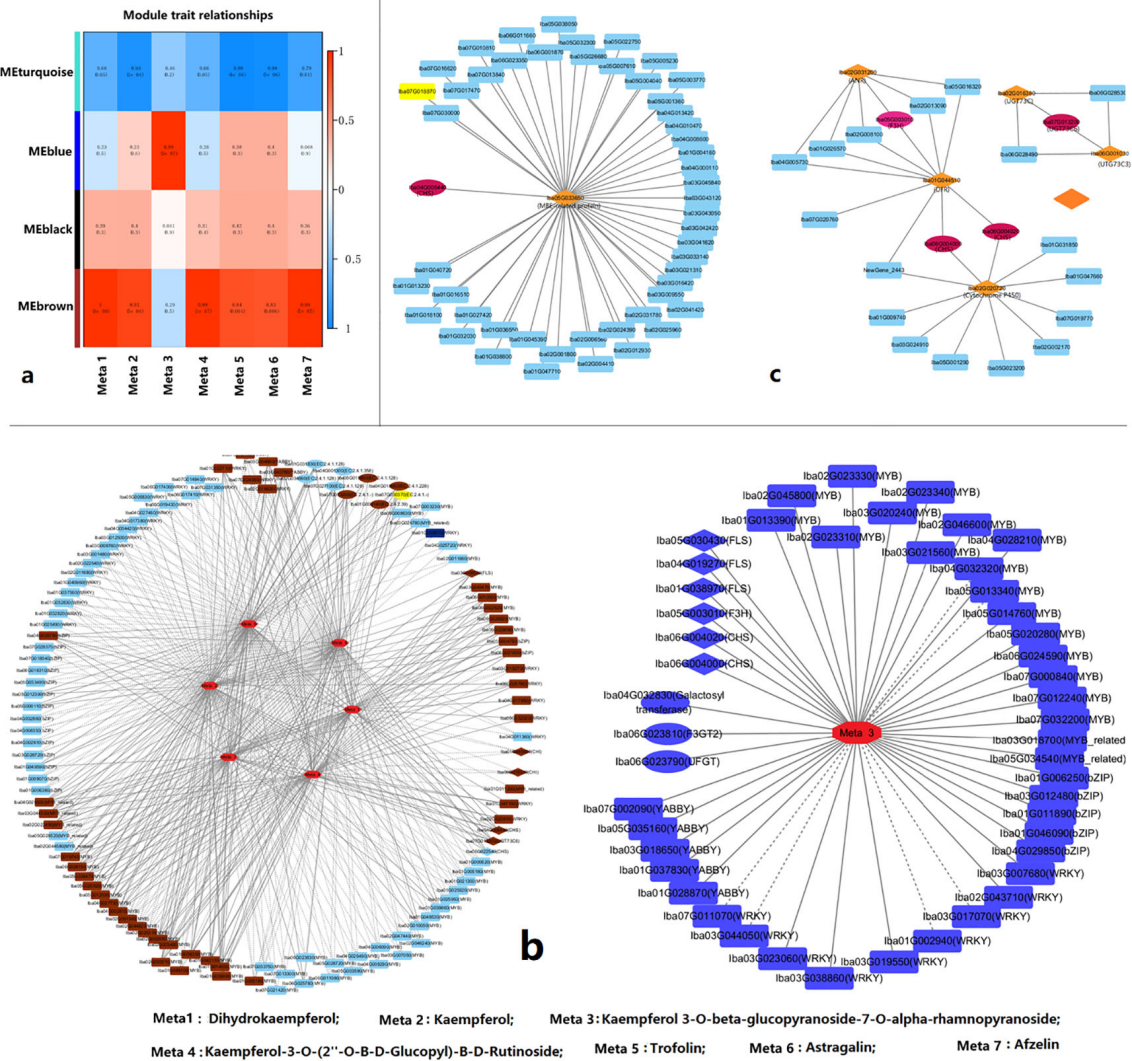
Simplified depiction of the regulatory network of kaempferol and kaempferol derivatives (KKDs) biosynthesis and regulation. **(a)** Trait-module correlations between KKDs and coexpressed gene network modules. **(b)** Correlated network between KKDs and putative key structural genes, TF genes, and glycosyltransferase genes. **(c)** Simplified depiction of the PPI network between structural genes and other genes that are at the first and second nodes.

To identify the genes putative responsible for the regulation of KKD biosynthesis in *I. balsamina*, WGCNA was performed using the expression profile data of transcriptomes from flowers, leaves, and roots, and dihydrokaempferol, kaempferol, kaempferol-3-O-β-glucopyranoside-7-O-α-rhamnopyranoside, astragalin, kaempferol-3-O-(2’’-O-β-D-glucopyl)-β-D-rutinoside, kaempferol (afzelin), and kaempferol-3-O-galactoside (trifolin) were used as traits for correlation analysis. Four gene coexpression network modules (GCENMs), designated as MEturquoise, MEblue, MEbrown, and MEblack, were identified, which included 2,641, 2,886, 1,872, and 43 genes, respectively (**Figure 5a**, **Dataset 12**). Along with kaempferol-3-O-β-glucopyranoside-7-O-α-rhamnopyranoside, which was positively correlated with MEblue, six other metabolites were significantly positively correlated with the MEbrown module but significantly negatively correlated with the MEturquoise module (**Figure 5a**, **Dataset 12**). These results suggest that the biosynthesis of kaempferol-3-O-β-glucopyranoside-7-O-α-rhamnopyranoside may be regulated by one gene network, whereas the other six metabolites are regulated by a different, yet related, gene network. Six, five, and one structural genes; 240, 160, and 244 TF genes; and 35, 33, and 31 glycosyltransferase genes were members of the MEblue, MEbrown, and MEturquoise modules, respectively (**Dataset 12**).

By integrating the results of the correlation analysis and WGCNA, several structural, TF, and glycosyltransferase genes that were both correlated with metabolites and located within modules were inferred to be putative key genes responsible for the organ-specific biosynthesis and regulation of KKD (**Figure 5b**, **Datasets 13**, **14**). Two *IbCHS* genes (*Iba06G004000* and *Iba06G004020*), one *IbF3H* gene (*Iba05G003010*), and three *IbFLS* genes (*Iba01G038970*, *Iba04G019270*, and *Iba05G030430*) were clustered into MEblue and were significantly positively correlated with kaempferol-3-O-β-glucopyranoside-7-O-α-rhamnopyranoside. Two *IbCHI* genes (*Iba01G040950* and *Iba06G018330*), one *IbCHI* gene (*Iba04G006440*), one *IbFLS* gene (*Iba03G008500*), and one *IbUGT73C6* gene (*Iba07G013200*) were clustered into MEbrown and were significantly correlated with three, four, or six of dihydrokaempferol, kaempferol, kaempferol-3-O-(2’’-O-β-D-glucopyl)-β-D-rutinoside, astragalin, trifolin, and keampferin (**Figure 5b**, **Datasets 13**, **14**). One IbCHS gene (*Iba06G022540*) was clustered into MEturquoise and was significantly negatively correlated with kaempferol, trifolin, and astragalin (**Figure 5b**, **Datasets 13**, **14**).

Among these correlated TF genes, 19 MYB, five YABBY, 13 WRKY, and eight bZIP genes were clustered into the MEblue module; 24 MYB, two YABBY, 10 WRKY, and three bZIP genes were clustered into the MEbrown module; and 25 MYB, 21 WRKY, and 13 bZIP family genes were clustered into the MEturquoise module (**Figure 5b**, **Datasets 13**, **14**). Twelve correlated glycosyltransferase genes that use flavonoid or flavonol as substrates were identified in three GCENMs. *Iba06G023790* (anthocyanidin 3-O-glucosyltransferase 2), *Iba06G023810* (anthocyanidin 3-O-glucosyltransferase 7), and *Iba04G032830* (xyloglucan 6-xylosyltransferase) were members of MEblue; *Iba05G015660* (scopoletin glucosyltransferase), *Iba07G013200* (flavonol-3-O-L-rhamnoside-7-O-glucosyltransferase), *Iba04G018680* (lactosylceramide 4-α-galactosyltransferase), and *Iba01G009140* (xyloglucan 6-xylosyltransferase) were members of MEbrown; and *Iba01G031830* (scopoletin glucosyltransferase), *Iba02G034660* (UDP-glucose flavonoid 3-O-glucosyltransferase 7), *Iba04G001300* (anthocyanidin 3-O-glucosyltransferase 2-like), Iba07G027100 (scopoletin glucosyltransferase), and *Iba07G000370* (galactan β–1,4-galactosyltransferase) were members of the MEturquoise module (**Datasets 13**, **14**).

A PPI analysis was performed between structural DEGs closely related to KKD biosynthesis and other expressed genes. The results showed that the IbCHS proteins encoded by *Iba06G004000* and *Iba06G004020* may directly interact with the cytochrome P450 encoded by *Iba02G020720*, the IbCHS protein encoded by *Iba04G006440* may directly interact with the MYB-like TF encoded by *Iba05G033650*, and the IbUGT73C6 encoded by *Iba07G013200* may directly interact with UDP-glucuronosyl and UDP-glucosyl transferases encoded by *Iba02G016380* and *Iba06G001030* (**Figure 5c**, **Dataset 15**). These results revealed that there are three different molecular regulatory mechanisms involved in KKD biosynthesis: interactions between structural gene-encoding proteins and structural proteins, interactions between structural proteins and P450, and interactions between structural proteins and TFs.

## 3 Discussion

### 3.1 Whole-genome duplication events may have significantly propelled the rapid cladogenesis of Balsaminaceae lineages and the explosive speciation of *Impatiens* species

The members of Balsaminaceae are grouped into two genera: *Hydrocera* and *Impatiens. Hydrocera* is a monotypic genus that contains only one species, *H. triflora*. *Impatiens* includes more than 900 species and is one of the largest genera among angiosperms [ (Song et al., 2003; Janssens et al., 2006, 2009). *Impatiens* species are rarely distributed in northern temperate regions and mostly occur in tropical and subtropical areas of the Old World (Grey-Wilson, 1980, 1985). Tropical Africa, South India, Sri Lanka, Madagascar, Southeast Asia, and the Sino-Himalaya have particularly high diversity (Mabberley, 2017). The genetic background of *Impatiens* species is highly complex, indicated by the wide range of somatic chromosome numbers among members (Singhal et al., 2017). *Impatiens* species also have highly variable floral morphology and color and have been described as “the dicot counterpart of orchids” (Janssens et al., 2012; Yuan et al., 2004). Studies have reported that Balsaminaceae lineages underwent rapid cladogenesis about 82 MYA, and the Balsaminaceae family recently diverged from its closest lineage, Tetrameristaceae, around 81 MYA (Rose et al., 2018). *Impatiens* and *Hydrocera* diverged from their common ancestor in the late Eocene (56–33.9 MYA), and *Impatiens s*pecies began diversifying in the early Miocene (5.3–23.3 MYA) (Janssens et al., 2009, 2012). Most *Impatiens* species occupy very narrow geographic ranges with small populations and are therefore endemic (Kumar and Sequiera, 1996; Bhaskar, 2006; Jyosna et al., 2009; Anil Kumar et al., 2011; Janssens et al., 2015). The cause of this rapid radiation speciation has not been investigated. Although a draft genome of *I. balsamina* has been deposited in GenBank (https://www.ncbi.nlm.nih.gov/datasets/taxonomy/63779/), the assembly is currently at the contig level and no analyses based on it have been reported. In this study, by analyzing the assembled draft genome of *I. balsamina*, evidence of two rounds of WGD events occurring about 87 MYA and 47 MYA was detected. Considering that WGD events are powerful speciation mechanisms (Marsit et al., 2021), it was proposed that these two rounds of WGD events greatly contributed, at least partially, to the rapid cladogenesis of Balsaminaceae lineages and the explosive speciation of *Impatiens* species.

### 3.2 A wide variety of secondary metabolites have been detected in the tissues of *I. balsamina*, providing valuable insights into its nutritional and medicinal applications

Among the beneficial SMs detected in this study, several compounds (e.g., kaempferol, astragalin, kaempferol-3-glucosylglucoside, quercetin, lawsone, 2-methoxy-1,4-naphthoquinone, cyanidin-3-O-galactoside (chloride), and fraxidin) have been reported in the tissues of *I. balsamina* previously (Qian et al., 2023), while most (e.g., piperlongumine, asperuloside, cyanidin 3-rutinoside, vasicinol, arteminin, and asperuloside) are reported in the tissues of *I. balsamina* for the first time. For both the previously known and newly detected SMs in the tissues of *I. balsamina*, their relative accumulation in the leaves, roots, and flowers of *I. balsamina* was examined, which offers reference value for the medicinal use of this species. Specifically, 2-methoxy-1,4-naphthoquinone (MNQ) is a representative beneficial compound of *I. balsamina* that accumulates at relatively high levels in the roots, leaves, and flowers. This differs slightly from the previous conclusion that MNQ accumulates mainly in the pericarps of *I. balsamina* (Foong et al., 2020). In addition to its medicinal value, *I. balsamina* is also consumed as a functional food. Its stems and flowers are eaten as vegetables, and its flowers are used in tea or wine beverages (Farnsworth and Bunyapraphatsara, 1992; Li et al., 2015). In this study, many amino acids and some vitamins (mainly vitamin B complexes) were detected in flowers. This finding may also provide useful information regarding the nutritional components of *I. balsamina.*

### 3.3 The integration of multi-omics data and comprehensive analyses has significantly deepened our understanding of the molecular pharmacognosy of *I. balsamina*

In recent decades, with advancements in high-throughput metabolomic and genome sequencing technologies, many SMs, including medically valuable compounds in diverse medicinal plants, have been identified through metabolomic analyses (Kang et al., 2022; Xiao et al., 2022). The molecular basis and genetic mechanisms, including the genes involved in the biosynthesis pathways of medically valuable SMs, have been identified by analyzing plant genomes and transcriptomes (Cheng et al., 2021b; Guo et al., 2021; Alami et al., 2022). Integrating multiple omics datasets is an effective approach to study the mechanisms of SM biosynthesis and regulation in medicinal plants (He et al., 2022; Burlat et al., 2023; Yang et al., 2023). This approach avoids limitations of single-omics analyses, as SMs are usually regulated by complex networks (Yang et al., 2023). For the large family of Impatiens, only limited omics information has been reported (Foong et al., 2019; Peng et al., 2021). For *I. balsamina*, only 2-methoxy-1,4-naphthoquinone, a beneficial SM with multiple medicinal effects and regulatory mechanisms, has been studied using comparative transcriptomics (Foong et al., 2020).

In this study, KKDs were investigated for their significant beneficial effects. In addition, the network involved in the biosynthesis of kaempferol and its direct derivatives, such as astragalin (kaempferol 3-O-glucoside), trifolin (kaempferol 3-O-β-D-galactopyranoside), and afzelin (synonyms: kaempferin, kaempferol 3-O-rhamnoside), was described. The enzymes CHI, CHS, F3H, and FLS sequentially catalyze the conversion of the precursor p-coumaryl-CoA into kaempferol. The CHI, CHS, F3H, and FLS genes have been identified in many plants (Calderón-Montaño et al., 2011; Santos-Buelga et al., 2019; Liu et al., 2021; Zhang et al., 2021; Jiang et al., 2023). Several glycosyltransferases (e.g., UGT736C) that catalyze the conversion of kaempferol into kaempferol derivatives have also been identified (Yonekura-Sakakibara and Saito, 2014; McIntosh and Owens, 2016; Ren et al., 2023). Studies have shown that the CHI, CHS, F3H, and FLS genes are regulated in different plant species by various transcription factors (TFs). These include MYB (Chen et al., 2023b), bZIP (Malacarne et al., 2016; Han et al., 2023), YABBY5 (Kayani et al., 2021), and WRKY (Wang et al., 2018). Additionally, miRNAs regulate the expression of the CHI, CHS, F3H, and FLS genes in some plant species (Yant et al., 2010; Dai et al., 2019; Lu et al., 2023).

In this study, by comparatively analyzing metabolomes, significant differences were observed in the accumulation levels of KKDs in the roots, leaves, and flowers of *I. balsamina*. By conducting functional annotation of protein-coding genes predicted from the assembled genome and comparing functional gene expression in the transcriptome, putative structural genes, including *IbCHI*, *IbCHS*, *IbF3H*, *IbFLS*, and *IbUGT736C*, in *I. balsamina* were identified; some were non-DEGs, while others were DEGs among leaves, roots, and flowers. These DEGs are inferred to be the key structural genes whose expression levels may directly influence the accumulation of KKDs in these organs. Additionally, correlation analysis and WGCNA were performed using data on the concentrations of dihydrokaempferol and six identified KKDs and gene expression profiles. Many genes and three GCENMs were found to be significantly correlated with one or more of the identified dihydrokaempferol and six KKDs. Among the correlated genes and modules, along with the abovementioned structural DEGs, some MYB, bZIP, WRKY, and YABBY family genes were identified as TFs, and these TF genes may be key candidates responsible for regulating KKD biosynthesis. Whether this regulation is positive or negative depends on the type of correlation. Moreover, some genes encoding glucosyltransferases that use flavonoids and flavonols as substrates were also found to be associated with the correlated genes and members of the correlated GCENMs.

Astragalin and trofolin were detected in the flowers of *I. balsamina* at very high concentrations, but paralogous genes encoding the glycosyltransferases flavonol 3-O-glucosyltransferase [EC:2.4.1.91] and kaempferol 3-O-beta-D-galactosyltransferase [EC:2.4.1.234] were not detected in the assembled genome sequence. Some studies have shown that glucosyltransferases in wild plants often exhibit high substrate heterogeneity (He et al., 2019; Zhang et al., 2022; Biswas and Thattai, 2020; Dai et al., 2018). It was speculated that homologs of genes encoding [EC:2.4.1.91] and [EC:2.4.1.234] may not be present in the genome of *I. balsamina*, and the corresponding functions are performed by other glucosyltransferase genes. For example, *Iba01G031830* encodes UDP-glucose flavonoid 3-O-glucosyltransferase 7 [EC:2.4.1.128], *Iba04G001300* encodes anthocyanidin 3-O-glucosyltransferase 2-like [EC:2.4.1.358], which may take over enzyme [EC:2.4.1.91] to catalyze the conversion of kaempferol into astragalin, and Iba04G018680 encodes galactan beta-1,4-galactosyltransferase GALS3-like [EC:2.4.1.-], which may take over enzyme [EC:2.4.1.234] to catalyze the conversion of kaempferol into trofolin. The expression of these genes is significantly correlated with KKD concentration and is part of the correlated GCENMs of KKDs. However, further molecular biology experiments are needed to verify which specific TF genes and glycosyltransferase genes directly regulate KKD synthesis. Meanwhile, WGCNA employed in this study is only considered an exploratory tool for hypothesis generation and candidate gene screening, and its conclusions require verification through future studies with larger sample sizes. Moreover, three putative different types of direct molecular regulatory mechanisms involved in KKD biosynthesis were observed through PPI analysis. The analysis in this study, based on multi-omics data, provides multiple insights into KKD biosynthesis in *I. balsamina.* Besides KKDs, other beneficial SMs can also be further described and analyzed using published genome, transcriptome, and metabolome data. Therefore, the resulting multi-omics data can greatly increase understanding of the molecular pharmacognosy of *I. balsamina*, which may help in the utilization of medicinal materials and drug production through genetic and metabolic engineering.

## 4 Conclusion

In this study, a 691.61 Mb chromosome-level draft genome of *I. balsamina* was assembled, with 302.57 Mb of repeat sequences and 32,949 protein-coding genes. Two rounds of WGD events were traced during the evolution of *I. balsamina* and were proposed to have played a key role in the radiation speciation of Balsaminaceae lineages and the genus *Impatiens*. The transcriptomes and metabolomes of roots, leaves, and flowers were also analyzed to determine the accumulation patterns of KKDs in different organs and to elucidate the molecular mechanisms involved in the biosynthesis and regulation of KKDs in *I. balsamina*. Many structural genes responsible for KKD biosynthesis were identified. Coexpressed gene network modules significantly correlated with one or more KKD genes, which include several structural genes, some WEKY, bHLH, and MYB family TF genes, and glycosyltransferase genes, were proposed to directly regulate KKD biosynthesis. Additionally, PPIs providing evidence of the molecular regulatory mechanisms of KKD biosynthesis were identified between structural genes and structural genes, between structural genes and p450 genes, and between structural genes and TF genes. To summarize, the findings in this study improved understanding of the genomic evolution and molecular pharmacognosy of *I. balsamina*. The multi-omics data generated and released also provide a framework for future breeding programs and pharmaceutical development, not only for *I. balsamina* but also for other *Impatiens* species.

## 5 Materials and methods

### 5.1 Extraction of total DNA and RNA

Total DNA was isolated from a young leaf of a cultivated I. balsamina plant with pink petals (coordinates: N 23°34′25″, E 116°28′56″; altitude: 654 m) using the Biomarker Plant DNA Kit (Biomarker Biotechnology Co., Ltd., Beijing, China). Total RNA was isolated from the young leaves, petals, and young roots of the same flowering plant using the Plant RNA Kit, following the manufacturer’s protocol (Omega Bio-Tek, Norcross, GA, USA). The extracted DNA and RNA were assessed for integrity using a Qubit fluorometer (Thermo Fisher Scientific, Waltham, MA, USA) and an Agilent 2100 Bioanalyzer with LabChip GX (Agilent Technologies, Santa Clara, CA, USA).

### 5.2 Library construction and sequencing

A 350 bp genomic sequencing library (genomic short-read library) was constructed following Illumina, Inc. protocols (San Diego, CA, USA). Specifically, genomic DNA was fragmented into pieces of about 350 bp using an ultrasonic oscillation device, and the broken segments underwent sticky-end repair, poly A addition, connector addition, target fragment selection, and PCR enrichment. Fragment size was checked via agarose gel electrophoresis, and the constructed 350 bp sequencing library was quantified using Qseq400 (BiOptic, New Taipei City, Taiwan, China) and Qubit™4.0 (Thermo Fisher Scientific, Waltham, MA, USA).

Construction of the SMRTbell library (genomic long-read library) was completed as follows. Double-stranded DNA fragments (6–20 kb long) were generated using a g-TUBE. Sticky ends were repaired and filled, and PacBio adapters were ligated to the DNA fragments. The DNA was purified, and any unconnected linkers were digested and removed using exonucleases. Sequencing primers were annealed, and DNA polymerase was added to form the PacBio sequencing library.

Hi-C library construction was as follows. Young leaves of sampled I. balsamina individuals were pretreated. Cell cross-linking was performed using formaldehyde to preserve interaction relationships and maintain the 3D structure between intracellular proteins and DNA, and between intracellular DNA and DNA. DNA was cleaved with the restriction endonuclease HindIII to produce sticky ends on both cross-linking sides. Biotin-labeled bases were added to repair the sticky ends, facilitating DNA purification and capture, and repaired DNA was cyclized. The cyclized DNA was decrosslinked and then purified into fragments of 300–700 bp, and strand-affinity magnetic beads were used to capture DNA fragments containing interaction relationships. The captured DNA was used for library construction.

The total RNA was used to construct the transcriptome sequencing library. First, mRNA enrichment using oligo(dT) magnetic beads was completed, and mRNA was randomly broken by adding fragmentation buffer. First-strand cDNA was synthesized using the fragmented mRNA as the template, and the second strand was synthesized using the first strand as the template. The cDNA was purified via agarose gel electrophoresis. After purification, double-stranded cDNA underwent end repair and A-tailing. It was then ligated with sequencing adapters, and fragment size was selected with AMPure XP beads (Beckman Coulter, Catalog Number A63880; Beckman Coulter, Brea, CA, USA). A cDNA library was obtained through PCR enrichment. The concentration of the cDNA library was checked using a Qubit™4.0 analyzer, and the inserted fragments were detected using a Qsep400 analyzer.

The HiSeq 4000 platform (San Diego, CA, USA) was used to sequence the genomic short-read, Hi-C, and RNA sequencing libraries through the PE150 module. For the RNA sequencing libraries, three biological replicates were performed on tissues from flowers, leaves, and roots. Raw short reads were removed from the poly G tail, and paired reads were further filtered if any single sequence was less than 100 bp, contained more than 10% of the bases identical to the following base, had more than 50% of bases with a quality score <10, or had a mean quality score <20. The remaining reads were used as clean data for subsequent analyses. The PacBio Sequel IIe platform (Pacific Biosciences Inc., Menlo Park, CA, USA) was used for long-read library sequencing to generate high-fidelity circular consensus sequence (CCS) data.

### 5.3 K-mer analysis

K-mer analysis for investigating genome size, ploidy, and heterozygosity was performed using Jellyfish 2.1.4 (-h 10000000000) (Kingsford, 2011) and GenomeScope 2.0 (-k 19 -p 2 -m 100000) (Ranallo-Benavidez et al., 2020).

### 5.4 Contig-level draft genome assembly and assessment

The CCS data were assembled into a primary contig-level draft genome using Hifiasm software (version 0.16) (I = 2, n = 4) (Cheng et al., 2021a). To evaluate assembly integrity, the primary contig-level draft genome was blasted against the CEGMA database, which includes 458 genes (Parra et al., 2007), and BUSCO v5.2.1, which includes 1614 genes (Simão et al., 2015). To assess assembly completeness, integrity, and coverage uniformity, clean short reads and CCS reads were realigned against the primary contig-level draft genome with BWA (Li and Durbin, 2009) and Minimap2 (https://github.com/lh3/minimap2) software, respectively.

### 5.5 Hi-C library applied in chromosome-level assembly of draft genome

After evaluating the clean Hi-C data, HiC-Pro software (v2.10.0) (https://github.com/nservant/HiC-Pro) was used to identify both valid and invalid interaction pairs. Valid interaction pairs were aligned against the primary contig-level draft genome with BWA software to identify mapped reads. To obtain valid mapped read data, the primary contig-level draft genome was used for grouping, sorting, and orienting the genomic sequences using LACHESIS (CLUSTER_MIN_RE_SITES = 27; CLUSTER_MAX_LINK_DENSITY = 2; ORDER_MIN_N_RES_IN_TRUNK = 8; ORDER_MIN_N_RES_IN_SHREDS = 8) (Li and Durbin, 2009), and then manual mapping and inspection were performed. Finally, a chromosome-level draft genome was obtained. After dividing the chromosome-level draft genome as 300 kp long into a bin, with the Hi-C read pair quantity that covered two individual bins being the intensity signal for the bin-bin interaction. This study adopted R 4.2.3 software (https://www.r-project.org/) for heatmap drawing. Circle diagrams displaying repeat sequence density, gene density, collinearity and GC content were drawn using Circle software (https://jokergoo.github.io/circlize/).

### 5.6 Annotation of the draft genome

The repeat elements, including interspersed repeats and tandem repeats, were detected from the draft chromosome genome. First, for interspersed repeats, RepeatModeler2 (v2.0.1) [46], where two ab initio prediction programs including RECON (v1.0.8) (http://eddylab.org/software/recon/) and RepeatScout (v1.0.6) (https://github.com/mmcco/RepeatScout) are utilized, was used, and RepeatClassifier (https://github.com/Dfam-consortium/RepeatModeler/blob/master/RepeatClassifier) was used for classifying prediction outcomes with the known database Dfam (v3.5) (https://ngdc.cncb.ac.cn/databasecommons/database/id/318).

Second, LTR_Retriever (version 2.9.0) (https://github.com/oushujun/LTR_retriever) was used for ab initio prediction of LTR retrotransposons. This tool primarily uses LTRharvest (version 1.5.10) (https://www.zbh.uni-hamburg.de/en/forschung/gi/software/ltrharvest.html) to identify LTR retrotransposons and can be used to analyze the prediction results from LTR_FINDER (version 1.07) (https://github.com/xzhub/LTR_Finder). The ab initio prediction outcomes were subsequently integrated based on known databases. Through this process, the dedicated repetitive sequence database personalized for a specific species was obtained. Finally, RepeatMask (v4.1.2) (http://repeatmasker.org/) was applied in predicting the genome transposon sequences (TEs) according to established repetitive sequence database. The MIcrosAtellite identification approach (MISA v2.1) (https://mybiosoftware.com/misa-microsatellite-identification-tool.html) and Tandem Repeat Finder (TRF, version 409, parameter: 27 7 80 10 50 500-h) (https://tandem.bu.edu/trf/trf.html) were adopted in predicting the tandem repeat sequences. We comprehensively predicted protein-coding genes in three ways. Specifically, Augustus (v3.1.0) (http://bioinf.uni-greifswald.de/augustus/) and SNAP (2006–07-28) (https://github.com/KorfLab/SNAP) were applied in ab initio prediction, GeMoMa (v1.7) (Keilwagen et al., 2019) was utilized in homology-based prediction, while prediction of expressed genes based on second-generation transcriptomes was based mostly on transcripts prepared through two methods. One method involved using Hisat [21] (v2.1.0) (http://ccb.jhu.edu/software/hisat/index.shtml) and StringTie (v2.1.4) (https://ccb.jhu.edu/software/stringtie/), and gene prediction was carried out with GeneMarkS-T (v5.1) (https://help.rc.ufl.edu/doc/GeneMarkS-T). Another method involved acquiring transcripts by means of Trinity (v2.11) (https://github.com/trinityrnaseq/trinityrnaseq/wiki) assembly, followed by PASA [25] (v2.4.1) (https://github.com/PASApipeline/PASApipeline) to predict genes. GMAP (2020 06 30) (Wu and Watanabe, 2005) was employed for comparing the third-generation transcriptome, which was subjected to several splice site treatments, followed by gene prediction using PASA (v2.4.1). At last, EVM (v1.1.1) (https://github.com/EVidenceModeler/EVidenceModeler) was applied in integrating our predicted outcomes acquired using these three approaches, with PASA (v2.4.1) being adopted to modify them. For protein-coding genes, their functional annotation was completed by querying databases below: NR (https://www.ncbi.nlm.nih.gov/refseq/), TrEMBL (https://www.uniprot.org/help/uniprotkb; https://www.bioinfo.pte.hu/more/TrEMBL.htm), eggNOG, KEGG (https://www.genome.jp/kegg/), KOG (http://www.ncbi.nlm.nih.gov/COG/), SWISS-PROT (http://www.expasy.ch/sprot), and Pfam (http://pfam.xfam.org/). Moreover, tRNAscan SE (v1.3.1) (https://github.com/tseemann/barrnap) and barrnap (v 0.9) (https://github.com/UCSC-LoweLab/tRNAscan-SE) were used to identify tRNAs and rRNAs, respectively. Additionally, prediction of miRNA snoRNAs and snRNAs was implemented with Rfam (v 14.5) database (https://rfam.org/) by Internal (v1.1) (http://eddylab.org/infernal/). The program GenBlastA (v1.0.4) (https://anaconda.org/bioconda/genblasta) was used for predicting pseudogenes.

### 5.7 Genome evolutionary dynamics analysis

The protein-coding genes encoding protein sequences (>100 amino acids long) of 12 species, including Trifolium pratense (https://ftp.ncbi.nlm.nih.gov/genomes/all/GCF/020/283/565/GCF_020283565.1_ARS_RC_1.1/), Linum usitatissimum (https://phytozome.jgi.doe.gov/pz/portal.html), Salix purpurea (https://phytozome-next.jgi.doe.gov/info/Spurpurea_v5_1), Sesamum indicum (https://doi.org/10.6084/m9.figshare.21151948), Coptis chinensis (https://ftp.ncbi.nlm.nih.gov/genomes/all/GCA/015/680/905/GCA_015680905.1_ASM1568090v1), Magnolia biondii (https://datadryad.org/stash/dataset/doi:10.5061/dryad.s4mw6m947), Gossypium Papaver somniferum (https://download.cncb.ac.cn/gwh/Plants/Papaver_somniferum_The_improved_chromosome-level_assembly_of_P._somniferum_GWHAZPJ00000000/), arboreum (https://ftp.ncbi.nlm.nih.gov/genomes/all/GCF/000/612/285/GCF_000612285.1_Gossypium_arboreum_v1.0/), Citrus sinensis (http://citrus.hzau.edu.cn/data/Genome_info/SWO.v3.0/), Ziziphus jujuba (https://ftp.ncbi.nlm.nih.gov/genomes/all/GCF/020/796/205/GCF_020796205.1_ASM2079620v1/), Litchi chinensis (https://data.mendeley.com/datasets/kggzfwpdr9/1), and I. balsamina were used to compare the genomes. Using Orthofinder v2.4 software (Emms and Kelly, 2019) with a 0.001 e-value and diamond alignment, protein sequences of 12 species were grouped into different gene families and their functions were annotated using the PANTHER v15 database (Mi et al., 2019).

An evolutionary tree of the above 12 species was built by IQ-TREE v1.6.11 (Nguyen et al., 2015) based on 1,067 homologous single-copy protein sequences. First, MAFFT v7.205 (Katoh et al., 2009) was adopted for aligning single-copy gene family sequences (parameters: –localpair –maxiterate 1000). Second, Gblocks v0.91b (Talavera and Castresana, 2007) (parameter: -b5 = h) was used to remove regions showing low or significantly divergent sequence alignment. Third, well-aligned gene family sequences for each species were connected end-to-end to obtain the supergene. Finally, ModelFinder (Kalyaanamoorthy et al., 2017) in IQ-TREE was utilized to detect the model.

For this evolutionary tree, M. biondii was set as the outgroup to obtain the rooted tree, and MCMCTREE of PAML v4.9i (Yang, 1997) was employed to determine bifurcation time. In addition, the TimeTree website (http://www.timetree.org/) was used to obtain fossil time for M. biondii vs. I. balsamina (151.6–170.1), L. chinensis vs. C. sinensis (68–85.4), I. balsamina vs. S. indicum (101.6–115.6), and P. somniferum vs. I. balsamina (126–132.4); this website was also used for subsequent correction of fossil time based on algorithm outputs.

Then, the MCMCTREE module in PAML was used to estimate required parameters for bifurcation time, gradient, and Hessian. At last, bifurcation time was estimated by a maximum likelihood approach using a correlated molecular clock and the JC69 model. The results were calculated twice to confirm consistency (the between-replicate correlation for the experiment was 1). The iteration number for the Markov chain was as follows: burn (discarded iterations) 500,000, sampfreq (sampling frequency) 30, and nsample (number of samples) 15,000,000. In the CAFE v4.2 software (Han et al., 2013), evolutionary trees of divergence times and gene family clustering results were used, and the gene family member quantity of each branch ancestor was estimated by the birth-mortality model to predict expansion and contraction of gene families compared with their ancestors. The standard for determining significant expansion and contraction was family-wide P-values and individual P-values <0.05. The resulting evolutionary tree with divergence times was illustrated with MCMCTreeR v1.1 (Puttick, 2019). GO and KEGG analyses were performed on species-specific and expansion/contraction gene families using ClusterProfile v3.4.4 (Yu et al., 2012).

Collinearity analysis was performed using Diamond v0.9.29.130 (Buchfink et al., 2015) to compare gene sequences between two species and determine collinear gene pairs (e < 1e–5, C-score >0.5, in which C-scores were screened with the JCVI software (https://github.com/tanghaibao/jcvi)). Based on the gff3 file, MCScanX (Wang et al., 2012) (parameter -m 15) was applied to analyze adjacency of similar gene pairs on chromosomes, ultimately obtaining genes within collinear blocks. Collinearity diagrams for linear patterns of different species were plotted using JCVI v0.9.13 (Tang et al., 2015).

The commonly used methods for identifying WGD are the Ks method and the fourfold synonymous (degenerative) third-codon transversion (4DTv) statistics. Ks analysis of paralogs was implemented with wgd v1.1.1 software (Zwaenepoel and Van de Peer, 2019), and 4DTv analysis was performed with scripts (https://github.com/JinfengChen/Scripts). The plot distributions for Ks and 4DTv values were drawn using the R (https://www.r-project.org/).

### 5.8 RNA sequencing data processing and analysis

The clean cDNA short reads were aligned onto the assembled final draft genome of I. balsamina by HISAT2 with the parameter settings -dta -p 6 -max-intronlen 5000000 (Kim et al., 2015b). StringTie (Pertea et al., 2015) was used to assemble mapped reads to reconstruct the transcriptome for subsequent analysis. The types of alternative splicing and associated expression in different samples were analyzed using ASprofile (Pertea et al., 2015). Boundaries of some annotated protein-coding genes in the reference draft genome were corrected, and some novel genes were assembled using this process.

Novel genes were annotated via BLAST against the Swiss-Prot, NR, COG, KOG, and KEGG databases with DIAMOND (Buchfink et al., 2015) and GO, InterProScan, InterPro, and Pfam databases using the HMMER software (Buchfink et al., 2015). The expression of each gene in the transcriptome was determined as follows: FPKM = cDNA Fragments/(Mapped Fragments (Millions) ∗ Transcript Length (kb)) (Trapnell et al., 2010). Pearson’s correlation coefficient (r) between the expression profiles of samples was used as an indicator of biological repeatability, and PCA of between-sample expression profiles was used to evaluate sample dispersion.

Gene expression differences between samples were analyzed according to FPKM values using DESeq2 software (Love et al., 2014). Thresholds for differentially expressed genes (DEGs) included fold change ≥2 and FDR <0.01.

### 5.9 Metabolome mass spectrometry

The same leaf, flower, and young root tissues used for RNA extraction were used for metabolome determination with the UPLC-ESI-MS/MS system (UPLC, Waters Acquity I-Class PLUS; MS, Applied Biosystems QTRAP 6500+), with three biological replicates for each tissue type. Samples were pretreated using the following steps: 30 s of vortexing, 10 min of grinding with steel balls, 10 min of sonication in an ice water bath, 1 h of standing at –20°C, 15 min of centrifugation (12,000 rpm, 4°C), transferring supernatants (500 μL) to an EP tube, drying supernatants in the vacuum concentrator, redissolving the dried extract in 160 μL acetonitrile:water (1:1) in a 2-mL injection bottle, and mixing a 10 μL sample with the QC sample for machine testing.

UPLC conditions included: Waters HSS-T3 column (1.8 μm, 2.1 mm × 100 mm), mobile phase A containing pure water with 0.1% formic acid/5 mM ammonium acetate, mobile phase B containing acetonitrile/0.1% formic acid, the elution process (initiated at 98% A, 2% B and held for 1.5 min, then linear gradient to 50% A, 50% B within 5.0 min, then linear gradient to 2% A, 98% B within 9.0 min and held for 1 min, eventually adjusted to 98% A, 2% B in 1 min and held for 3 min), column oven temperature of 50°C, injection volume of 2 μL, and flow rate of 0.35 mL/min.

The effluent was connected to the ESI-triple quadrupole-linear ion trap-MS under the following parameters: source temperature 550°C, ion spray voltage 5500 V/–4500 V (positive/negative ion modes), ion source gas I, gas II, and curtain gas at 50, 55, and 35 psi, collision-activated dissociation (medium), mass calibration (100 μmol/L polypropylene glycol solutions under LIT mode), and instrument tuning (10 μmol/L polypropylene glycol solutions in QQQ). QQQ scans were obtained through multiple reaction monitoring (MRM) experiments using collision gas (nitrogen) (medium). For each MRM transition, collision energy (CE) and declustering potential (DP) were carefully optimized. Monitoring of specific MRM transitions was conducted for each time period according to metabolite elution. Dichlorophenylalanine and deuterated cholic acid (1 ppm each) served as internal standards in the above process.

### 5.10 Qualitative, quantitative, and metabolomic analyses

Metabolites were identified based on secondary spectral information by searching the database GB-PLANT, constructed by Biomarker Technologies, Inc. (http://www.biomarker.com.cn/). Isotope signals, repetitive signals containing K+, NH4+, Na+ ions, and large-molecular-weight fragment ions were eliminated from analyses. Metabolites were quantified with triple quadrupole mass spectrometry under MRM.

After acquiring mass spectrometry data of metabolites from different samples, peak area integrations were performed for all MS peaks, and integral calibration was conducted for the peaks of the same metabolite across different samples. Specifically, the peak areas were standardized to the total peak area, and the relative MS peak area of each metabolite corresponds to its relative concentration. Spearman’s correlation analysis and principal component analysis were first performed on mass spectrometry data to assess sample repeatability and control sample quality.

Classification and pathway data of detected metabolites were searched in KEGG databases. Content fold change (FC) of each metabolite was determined according to grouping data, and corresponding P-values were estimated using t-tests to assess significance. OPLS-DA modeling was implemented with the R package ropls (https://bioconductor.uib.no/packages/3.20/bioc/html/ropls.html), and 200 permutation tests were performed to verify model reliability. Model VIP values were calculated via multiple cross-validation. P-value, FC, and VIP value for the OPLS-DA model were used together for screening differentially accumulated metabolites (DAMs) with log2FC >1, P <0.05, and VIP >1. Significant DAMs associated with KEGG pathways were evaluated by the hypergeometric distribution test.

### 5.11 Combined analyses of transcriptome and metabolome data

Correlations of DEGs and DAMs detected for each comparison group, i.e., flower vs. root, root vs. leaf, and leaf vs. flower, were analyzed with the cor program of R (https://cran.r-project.org/). DEG–DAM pairs with Pearson’s correlation coefficients > 0.8 (p < 0.05) were subjected to further analysis. DEG–DAM pairs annotated in the same KEGG pathway were mapped to a network diagram to represent the correlation. A bar chart was drawn to display the enrichment degree of pathways enriched with DAMs and DEGs. DEGs and DAMs in each pathway were subjected to canonical correlation analysis (CCA), and four regions were identified using a cross plot.

### 5.12 Coexpression gene network analysis

Weighted gene correlation network analysis (WGCNA) based on leaf, flower, and root transcriptome expression profiles and the contents of seven metabolites (dihydrokaempferol, kaempferol, and five kaempferol derivatives, including kaempferol 3-O-β-glucopyranoside-7-O-α-rhamnopyranoside, kaempferol 3-O-glucoside (astragalin), keampferin (afzelin), kaempferol-3-O-galactoside (trofolin), and kaempferol-3-O-(2″-O-β-D-glucopyl)-β-D-rutinoside) was performed using the WGCNA R (Langfelder and Horvath, 2008) built into an online tool package (https://international.biocloud.net/zh/software/tools). The thresholds |CC| >0.80 and P <0.05 were used for significant correlations between traits and modules.

Protein-protein interactions (PPIs) of identified DEGs were predicted with the STRING database (http://string-db.org/). Specifically, BLAST software was employed to conduct sequence alignment between the target genes and the proteins in the database for homologous protein identification. The interaction network was constructed based on the interaction relationships of the identified homologous proteins, with a confidence score threshold set at 150 or higher. Cytoscape software was employed to visualize the direct correlation network between expressed genes and the seven metabolites, as well as the PPIs of the DEGs (Shannon et al., 2003).

## Data Availability

The datasets presented in this study can be found in online repositories. The names of the repository/repositories and accession number(s) can be found in the article/**Supplementary Material**.

## Supplementary material

Supplementary Data Sheet 1 Integrated functional annotation for predicting protein-coding genes.

Supplementary Data Sheet 2 Gene family classification for predicting protein-coding genes.

Supplementary Data Sheet 3 Data regarding Single-copy genes encoding protein sequences used to construct phylogenetic trees.

Supplementary Data Sheet 4 Data regarding duplicated gene pairs.

Supplementary Data Sheet 5 Data regarding the Ks values of all collinear genes.

Supplementary Data Sheet 6 Data regarding metabolites in roots, flowers, and leaves.

Supplementary Data Sheet 7 Data regarding differentially accumulated metabolites among roots, flowers, and leaves.

Supplementary Data Sheet 8 Data regarding the expression of genes in roots, flowers, and leaves.

Supplementary Data Sheet 9 Listing of glycosyltransferase genes and transcription factor genes in roots, flowers, and leaves.

Supplementary Data Sheet 10 Data regarding the differentially expressed genes among roots, flowers, and leaves.

Supplementary Data Sheet 11 Data regarding expression correlation between genes and KKDs.

Supplementary Data Sheet 12 Four coexpressed gene network modules significantly correlated with one or more KKDs.

Supplementary Data Sheet 13 Data concerning structural genes and transcription factor genes encoding glycosyltransferase genes that are correlated with seven metabolites and clustered into coexpression network modules.

Supplementary Data Sheet 14 Data regarding the correlation between KKDs and genes encoding transcription factors and glycosyltransferases, which are involved in coexpression network modules.

Supplementary Data Sheet 15 Protein-protein interaction information between differentially expressed gene.
