# Supplementary material for: A chromosome level genome, as well as transcriptomes and metabolomes, insights into genome evolution and the biosynthesis of kaempferol and kaempferol derivatives in Impatiens balsamina (Balsaminaceae)
Source: Front Plant Sci. 2026 Feb 11;17:1725789. doi: 10.3389/fpls.2026.1725789 (PMC12932442; doi:10.3389/fpls.2026.1725789)
Supplement: Supplementary Data Sheet 1 — Integrated functional annotation for predicting protein-coding genes. [file DataSheet1.zip › all supplementary files/Supplementary tables.docx]

Table S1 Summary of genomic short-read data for *Impatiens balsamina*

| Items | Counts |
| --- | --- |
| Total Data (Gb) | 61.54 |
| Filtered data (Gb) | 53.44 |
| Depth (×) | 101.80 |
| GC (%) | 32.31 |
| Q20 (%) | 99.47 |
| Q30 (%) | 96.81 |

Table S2 Summary of circular consensus sequencing (CCS) data for *I. balsamina*

| Items | Counts |
| --- | --- |
| Reads Number | 1,948,178 |
| Reads Base | 33,246,751,629 bp |
| N50 of Reads Length | 17,464 bp |
| Mean Reads Length | 17,066 bp |
| Max Read Length | 49,723 bp |

Table S3 Summary of the draft genome of *I. balsamina* at the contig level

| Items | Counts |
| --- | --- |
| Contig Number | 405 |
| Contig Length | 691,608,837 bp |
| N50 of Contig length | 81,302,920 bp |
| N90 of Contig length | 27,418,586 bp |
| Max of Contig length | 102,387,436 bp |
| GC content | 33.59 % |

Table S4 Mapped statistics for realigning the short-read and long-read sequences against the contig-level draft genome of *I. balsamina*

| Data Type | Short Reads Data | CCS Data |
| --- | --- | --- |
| Total reads | 414,769,436 | 1,948,178 |
| Mapped reads | 409,572,094 | 1,933,275 |
| Mapped (%) | 98.75% | 99.24% |
| Coverage ratio (1X) | 99.93% | 99.94% |
| Coverage ratio (5X) | 99.83% | 99.6% |
| Coverage ratio (10X) | 99.69% | 99.43% |
| Coverage ratio (20X) | 99.14% | 99.04% |

Table S5 Summary of the Hi-C assembled data

| Items | Counts |
| --- | --- |
| Total reads | 204,097,719 |
| Total bases | 61,077,887,896 bp |
| GC % | 34.99 |
| Q20 % | 99.41 |
| Q30 % | 96.03 |
| Total read pairs | 204,097,719 |
| Mapped read | 373,820,682 |
| Unique mapped read pairs | 174,183,373 |
| Valid Interaction Pairs | 108,206,138 (62.12%) |
| Dangling End Pairs | 53201125 (30.54%) |
| Re-ligation Pairs | 3,450,012 (1.98%) |
| Self-cycle Pairs | 276,947 (0.16%) |
| Dumped Pairs | 9,049,151 (5.20%) |

Table S6 Summary of the Hi-C assembly

| Items | Counts |
| --- | --- |
| Scaffold Number | 399 |
| Scaffold Length | 691,609,437 bp |
| N50 of Scaffold | 96,691,550 bp |
| N90 of Scaffold | 63,005,857 bp |
| Max Scaffold | 119,059,492 bp |

Table S7 Summary of chromosome mounting

| Group | Cluster Number | Cluster  Length | Order Number | Order  Length |
| --- | --- | --- | --- | --- |
| Chr01 | 16 | 122,051,594 bp | 3 | 119,059,292 bp |
| Chr02 | 9 | 110,169,374 bp | 2 | 109,877,160 bp |
| Chr03 | 3 | 107,892,591 bp | 2 | 107,839,097 bp |
| Chr04 | 13 | 97,279,484 bp | 1 | 96,691,550 bp |
| Chr05 | 5 | 93,445,219 bp | 2 | 93,208,727 bp |
| Chr06 | 1 | 81,302,920 bp | 1 | 81,302,920 bp |
| Chr07 | 3 | 63,088,362 bp | 2 | 63,005,757 bp |
| Total | 50 | 675,229,544 bp | 13 | 670,984,503 bp |
| Ratio | 12.35% | 97.63% | 26.00% | 99.37% |

Table S8 Statistics of repetitive elements in the *I. balsamina* genome

| Type | Number | Length (bp) | Rate (%) |
| --- | --- | --- | --- |
| ClassI:Retroelement | 299,439 | 302,565,441 | 43.75 |
| ClassI/LINE | 32,587 | 15,102,334 | 2.18 |
| ClassI/LTR/Caulimovirus | 302 | 395,896 | 0.06 |
| ClassI/LTR/Copia | 124,487 | 179,490,640 | 25.95 |
| ClassI/LTR/ERV | 1,450 | 128,957 | 0.02 |
| ClassI/LTR/Gypsy | 9,759 | 9,651,182 | 1.40 |
| ClassI/LTR/Ngaro | 957 | 170,532 | 0.02 |
| ClassI/LTR/Pao | 40 | 2,963 | 0 |
| ClassI/LTR/Unknown | 124,581 | 94,764,342 | 13.70 |
| ClassI/SINE | 5,276 | 2,858,595 | 0.41 |
| ClassII:DNA transposon | 145,033 | 53,003,629 | 7.66 |
| ClassII/CACTA | 12,578 | 7,777,301 | 1.12 |
| ClassII/Crypton | 74 | 3,846 | 0 |
| ClassII/Dada | 386 | 21,165 | 0 |
| ClassII/Ginger | 157 | 8,217 | 0 |
| ClassII/Helitron | 737 | 261,090 | 0.04 |
| ClassII/IS3EU | 41 | 2,212 | 0 |
| ClassII/Kolobok | 721 | 60,241 | 0.01 |
| ClassII/Maverick | 29 | 2,697 | 0 |
| ClassII/Merlin | 308 | 17,640 | 0 |
| ClassII/Mutator | 5,604 | 3,485,906 | 0.5 |
| ClassII/P | 205 | 13,832 | 0 |
| ClassII/PIF-Harbinger | 2,767 | 1,141,775 | 0.17 |
| ClassII/PiggyBac | 183 | 8,323 | 0 |
| ClassII/Tc1-Mariner | 807 | 178,496 | 0.03 |
| ClassII/Unknown | 114,612 | 37,391,533 | 5.41 |
| ClassII/Zisupton | 12 | 536 | 0 |
| ClassII/hAT | 5,812 | 2,628,819 | 0.38 |
| Unknown | 15 | 829 | 0 |
| Total | 444,487 | 355,569,899 | 51.41 |

Table S9 Summary of tandem repeats in the *I. balsamina* genome

| Type | Number | Length (bp) | Rate (%) |
| --- | --- | --- | --- |
| Microsatellite(1-9 bp units) | 192,827 | 3,528,743 | 0.51 |
| Minisatellite(10-99 bp units) | 196,360 | 16,669,260 | 2.41 |
| Satellite(>=100 bp units) | 20,838 | 108,521,439 | 15.69 |
| Total | 410,025 | 128,719,442 | 18.61 |

Table S10 Summary of the predicted protein-coding genes

| Method | Software | Species | Gene number |
| --- | --- | --- | --- |
| Ab initio | Augustus | - | 30,806 |
|  | SNAP | - | 65,671 |
| Homology-based | GeMoMa | A. thaliana | 25,357 |
|  |  | C. sinensis | 25,793 |
|  |  | L. usitatissimum | 24,921 |
|  |  | S. indicum | 25,330 |
| RNAseq | GeneMarkS-T | - | 18,828 |
|  | PASA | - | 21,132 |
| Integration | EVM | - | 32,949 |

Table S11 Integrity evaluation of the prediction of protein-coding genes via BLAST searches against the BUSCO database

| Items | Counts |
| --- | --- |
| Complete BUSCOs(C) | 1581 (97.96%) |
| Complete and single-copy BUSCOs(S) | 1489 (92.26%) |
| Complete and duplicated BUSCOs(D) | 92 (5.70%) |
| Fragmented BUSCOs(F) | 12 (0.74%) |

Table S12 Annotation statistics of the predicted protein-coding genes

| Annotated Database | Annotated Number | Annotated Ratio (%) |
| --- | --- | --- |
| GO_Annotation | 25,455 | 77.26 |
| KEGG_Annotation | 23,049 | 69.95 |
| KOG_Annotation | 16,770 | 50.90 |
| Pfam_Annotation | 26,133 | 79.31 |
| Swissprot_Annotation | 24,888 | 75.53 |
| TrEMBL_Annotation | 30,775 | 93.40 |
| eggNOG_Annotation | 26,051 | 79.06 |
| nr_Annotation | 30,461 | 92.45 |
| All_Annotated | 30,938 | 93.90 |

Table S13 Summary of non-coding RNA genes and pseudogenes

| Item | Counts |
| --- | --- |
| rRNA | 11,984 |
| tRNA | 3,889 |
| miRNA | 27 |
| snRNA | 71 |
| snoRNA | 58 |
| Pseudogene | 277 |

Table S14 Summary of the gene families of 12 species

| Items | Cchine  *Coptis chinensis* | Csinen*Citrus sinensis* | Garbor  *Gossypium arboreum* | Ibalsa | Lchine *Litchi chinensis* | Lusita  *Linum usitatissimum* | Mbiond  *Magnolia biondii* | Psomni  *Papaver somniferum* | Sindic*Sesamum indicum* | Spurpu  *Salix purpurea* | Tprate  *Trifolium pratense* | Zjuju  *Ziziphus jujuba* |
| --- | --- | --- | --- | --- | --- | --- | --- | --- | --- | --- | --- | --- |
| Number of genes | 36,637 | 29,769 | 33,937 | 32,949 | 58,121 | 43,471 | 38,602 | 55,316 | 24,261 | 34,537 | 33,272 | 25,295 |
| Number of genes in orthogroups | 27,101 | 22,739 | 32,025 | 28,439 | 51,631 | 37,226 | 29,165 | 44,512 | 20,150 | 32,289 | 30,952 | 23,887 |
| Number of unassigned genes | 9,536 | 7,030 | 1,912 | 4,510 | 6,490 | 6,245 | 9,437 | 10,804 | 4,111 | 2,248 | 2,320 | 1,408 |
| Percentage of genes in orthogroups | 74.00 | 76.40 | 94.40 | 86.30 | 88.80 | 85.60 | 75.60 | 80.50 | 83.10 | 93.50 | 93.00 | 94.40 |
| Number of orthogroups containing species | 15,865 | 17,012 | 17,240 | 16,082 | 18,328 | 17,030 | 14,080 | 17,635 | 14,107 | 17,141 | 16,539 | 16,655 |
| Number of species-specific orthogroups | 2,094 | 949 | 647 | 892 | 1,621 | 1,350 | 1,862 | 2,943 | 739 | 694 | 1,161 | 421 |
| Number of genes in species-specific orthogroups | 6,911 | 3,022 | 3,525 | 3,735 | 5,506 | 4,243 | 9,616 | 10,068 | 2,511 | 3,454 | 5,744 | 1,768 |

Table S15 Summary of metabolites in the flowers, roots, and leaves of *I. balsamina*

| Class I | Class II | Number of Metabolites | | | |
| --- | --- | --- | --- | --- | --- |
|  |  | Total | Flower | Root | Leaf |
| Amino acids | Alpha Amino Acids | 5 | 5 | 5 | 5 |
|  | Dipeptide | 4 | 4 | 4 | 4 |
|  | Other | 75 | 75 | 72 | 74 |
|  | Peptides | 15 | 15 | 15 | 14 |
|  | total | 99 | 99 | 96 | 97 |
| Phenylpropanoids | Phenylpropanols | 3 | 3 | 3 | 3 |
|  | Simple Phenylpropanols | 5 | 5 | 4 | 5 |
|  | Other | 13 | 13 | 8 | 13 |
|  | Cinnamic Acids And Derivatives | 2 | 2 | 1 | 2 |
|  | Total | 23 | 23 | 16 | 23 |
| Polyphenols | Phenols | 4 | 4 | 4 | 4 |
|  | Monophenols | 9 | 8 | 9 | 9 |
|  | Phenolic Acids | 6 | 6 | 6 | 6 |
|  | Other | 27 | 25 | 24 | 26 |
|  | Bisphenol | 1 | 1 | 1 | 1 |
|  | Glycosides | 2 | 2 | 2 | 2 |
|  | Total | 49 | 46 | 46 | 48 |
| Nucleotides | Ribonucleotides | 6 | 6 | 6 | 6 |
|  | Flavin Nucleotides | 1 | 1 | 1 | 1 |
|  | Pyrimidine Nucleosides | 7 | 7 | 6 | 7 |
|  | Purine Nucleosides | 14 | 14 | 12 | 14 |
|  | Other | 6 | 6 | 6 | 6 |
|  | Deoxyribonucleosides | 3 | 3 | 3 | 3 |
|  | Total | 37 | 37 | 34 | 37 |
| Flavonoids | Chalcones | 3 | 2 | 3 | 2 |
|  | Monophenols | 2 | 2 | 2 | 2 |
|  | Polyphenols | 26 | 25 | 22 | 23 |
|  | Flavonones | 2 | 2 | 2 | 2 |
|  | Flavanonols | 2 | 2 | 2 | 2 |
|  | Anthocyanins | 4 | 4 | 4 | 4 |
|  | Flavonols | 21 | 19 | 17 | 21 |
|  | Flavonoid Glycosides | 2 | 2 | 0 | 2 |
|  | Other | 26 | 26 | 23 | 23 |
|  | Hydroxyflavone | 2 | 2 | 2 | 2 |
|  | Biflavones | 2 | 2 | 2 | 2 |
|  | Glycosides | 8 | 8 | 7 | 7 |
|  | Isoflavones | 6 | 5 | 5 | 6 |
|  | Total | 106 | 101 | 91 | 98 |
| Quinones | Benzene Quinones | 2 | 2 | 2 | 2 |
|  | Anthraquinones | 6 | 6 | 4 | 5 |
|  | Naphthoquinones | 4 | 4 | 4 | 4 |
|  | Other | 3 | 3 | 2 | 3 |
|  | Total | 15 | 15 | 12 | 14 |
| Others | Benzofurans | 1 | 1 | 1 | 1 |
|  | Pyridine And Its Derivatives | 1 | 1 | 1 | 1 |
|  | Choline | 1 | 1 | 1 | 1 |
|  | Furans | 1 | 1 | 1 | 1 |
|  | Glucosinolate | 1 | 1 | 1 | 1 |
|  | Purines | 1 | 1 | 1 | 1 |
|  | Other | 45 | 44 | 42 | 44 |
|  | Glycosides | 2 | 2 | 2 | 2 |
|  | Total | 53 | 52 | 50 | 52 |
| Alkaloids | Pyridine Alkaloids | 7 | 6 | 5 | 7 |
|  | Pyrrolizidine Alkaloids | 1 | 1 | 1 | 1 |
|  | Anthranilic Acid Alkaloids | 1 | 1 | 1 | 1 |
|  | Quinoline Alkaloids | 3 | 3 | 3 | 3 |
|  | Lysine Alkaloids | 2 | 2 | 1 | 2 |
|  | Tyrosine Alkaloids | 1 | 1 | 1 | 1 |
|  | Tropane Alkaloids | 2 | 2 | 1 | 2 |
|  | Ornithine Alkaloids | 4 | 4 | 4 | 3 |
|  | Piperidine Alkaloids | 3 | 3 | 3 | 2 |
|  | Other | 48 | 44 | 43 | 43 |
|  | Solanaceous Alkaloids | 1 | 1 | 1 | 1 |
|  | Tropane Alkaloid | 1 | 1 | 0 | 1 |
|  | Tryptophan Alkaloids | 3 | 3 | 3 | 2 |
|  | Betalain | 2 | 2 | 2 | 2 |
|  | Terpenoid Alkaloids | 1 | 1 | 1 | 1 |
|  | Berberine Alkaloids | 1 | 0 | 1 | 0 |
|  | Nicotinic Acid Alkaloids | 1 | 1 | 1 | 1 |
|  | Isoquinoline Alkaloids | 5 | 3 | 4 | 5 |
|  | Indole Alkaloids | 5 | 4 | 5 | 5 |
|  | Total | 92 | 83 | 81 | 83 |
| Sugars and alcohols | Amino Alcohols | 5 | 5 | 3 | 5 |
|  | Amino Sugars | 1 | 1 | 1 | 1 |
|  | Monosaccharides | 14 | 14 | 13 | 13 |
|  | Polysaccharides | 3 | 3 | 3 | 3 |
|  | Polyol | 5 | 5 | 5 | 5 |
|  | Disaccharides | 6 | 6 | 6 | 6 |
|  | Hexosephosphates | 3 | 3 | 3 | 3 |
|  | Glucans | 1 | 1 | 1 | 1 |
|  | Other | 9 | 9 | 9 | 7 |
|  | Sugar Alcohols | 8 | 8 | 7 | 8 |
|  | Glycosides | 8 | 7 | 4 | 8 |
|  | Sugar Acids | 3 | 3 | 3 | 3 |
|  | Glycols | 1 | 1 | 1 | 1 |
|  | Fatty Alcohol | 2 | 1 | 1 | 2 |
|  | Secondary Alcohol | 1 | 1 | 0 | 1 |
|  | Total | 70 | 68 | 60 | 67 |
| Terpenoids | Sesquiterpenoid | 13 | 13 | 12 | 11 |
|  | Monoterpenes | 8 | 8 | 7 | 8 |
|  | Diterpene | 17 | 16 | 16 | 15 |
|  | Iridoids | 10 | 10 | 9 | 9 |
|  | Other | 4 | 3 | 3 | 4 |
|  | Triterpenes | 18 | 15 | 16 | 16 |
|  | Tetraterpenoid | 1 | 1 | 1 | 1 |
|  | Total | 71 | 66 | 64 | 64 |
| Ketones, Aldehydes, Acids | Phenylpropanoic Acids | 1 | 1 | 1 | 1 |
|  | Benzaldehydes | 5 | 5 | 5 | 5 |
|  | Acetophenones | 1 | 1 | 1 | 1 |
|  | Aryl-Aldehydes | 3 | 3 | 2 | 3 |
|  | Polyketides | 2 | 2 | 2 | 2 |
|  | Other | 15 | 15 | 15 | 15 |
|  | Chromones | 1 | 1 | 1 | 1 |
|  | Sugar Acids | 4 | 4 | 4 | 4 |
|  | Pentanones | 1 | 1 | 0 | 0 |
|  | Total | 33 | 33 | 31 | 32 |
| Vitamins | Other | 2 | 2 | 2 | 2 |
|  | Vitamin B Complex | 5 | 5 | 5 | 5 |
|  | Total | 7 | 7 | 7 | 7 |
| Coumarins | Benzopyrans | 1 | 0 | 1 | 0 |
|  | Other | 25 | 25 | 24 | 22 |
|  | Hydroxycoumarins | 2 | 2 | 2 | 2 |
|  | Coumaric Acids | 1 | 1 | 1 | 0 |
|  | Isocoumarins | 1 | 1 | 1 | 1 |
|  | Total | 30 | 29 | 29 | 25 |
| Organic acid | Benzoic Acids | 3 | 3 | 2 | 3 |
|  | Benzoates | 3 | 3 | 3 | 3 |
|  | Aromatic Organic Acids | 2 | 2 | 2 | 2 |
|  | Total | 8 | 8 | 7 | 8 |
| Xanthones | Other | 4 | 3 | 2 | 4 |
| Organic acid | Other | 13 | 12 | 12 | 12 |
|  | Carboxylic Acids | 57 | 57 | 45 | 53 |
|  | Toral | 70 | 69 | 57 | 65 |
| Steroids | Hormone | 1 | 1 | 1 | 1 |
|  | Other | 4 | 4 | 4 | 4 |
|  | Glycosides | 1 | 1 | 1 | 1 |
|  | Phytosterols | 3 | 3 | 3 | 3 |
|  | Total | 9 | 9 | 9 | 9 |
| Lipid | Glycerophospholipids | 3 | 3 | 3 | 3 |
|  | Glycerol Ester | 2 | 2 | 2 | 2 |
|  | Phospholipids | 1 | 1 | 1 | 1 |
|  | Other | 2 | 2 | 2 | 2 |
|  | Sphingolipid | 4 | 4 | 3 | 4 |
|  | Free Fatty Acids | 1 | 1 | 1 | 1 |
|  | Fatty Alcohol | 1 | 1 | 1 | 1 |
|  | Fatty Acids | 10 | 10 | 10 | 9 |
|  | Fatty Acyls | 28 | 28 | 27 | 26 |
|  | Fatty Amides | 2 | 2 | 2 | 2 |
|  | Total | 54 | 54 | 52 | 51 |
| ALL |  | 828 | 795 | 743 | 782 |

Table S16 Summary of RNA sequencing data

| Samples | Number of Clean Reads | Clean Bases (bp) | GC Content | %≥Q30 |
| --- | --- | --- | --- | --- |
| Root-1 | 20,183,341 | 6,042,424,916 | 45.28% | 93.20% |
| Root-2 | 20,019,355 | 5,993,206,676 | 45.19% | 93.44% |
| Root-3 | 19,898,924 | 5,957,173,602 | 45.24% | 93.25% |
| Flower-1 | 21,463,947 | 6,425,244,016 | 45.70% | 93.28% |
| Flower-2 | 20,506,801 | 6,140,533,442 | 44.95% | 93.37% |
| Flower-3 | 20,457,500 | 6,124,939,414 | 44.90% | 93.30% |
| Leaf-1 | 22,027,903 | 6,586,544,468 | 46.37% | 94.19% |
| Leaf-2 | 23,915,606 | 7,141,733,018 | 45.75% | 94.42% |
| Leaf-3 | 21,048,626 | 6,278,096,622 | 45.45% | 94.77% |

Table 17 Mapping statistics of RNA sequencing reads against the reference genome

| Sample | Total Reads | Mapped Reads | Uniq Mapped Reads | Multiple Map Reads | Reads Map to '+' | Reads Map to '-' |
| --- | --- | --- | --- | --- | --- | --- |
| Root-1 | 40,366,682 | 38,309,608 (94.90%) | 37,420,368 (92.70%) | 889,240 (2.20%) | 19,795,797 (49.04%) | 19,824,794 (49.11%) |
| Root-2 | 40,038,710 | 38,029,453 (94.98%) | 37,150,600 (92.79%) | 878,853 (2.20%) | 19,646,814 (49.07%) | 19,674,740 (49.14%) |
| Root-3 | 39,797,848 | 37,802,082 (94.99%) | 36,939,929 (92.82%) | 862,153 (2.17%) | 19,519,179 (49.05%) | 19,550,513 (49.12%) |
| Flower-1 | 42,927,894 | 35,502,902 (82.70%) | 34,570,598 (80.53%) | 932,304 (2.17%) | 18,447,210 (42.97%) | 18,464,579 (43.01%) |
| Flower-2 | 41,013,602 | 37,868,963 (92.33%) | 36,964,347 (90.13%) | 904,616 (2.21%) | 19,581,617 (47.74%) | 19,590,317 (47.77%) |
| Flower-3 | 40,915,000 | 37,647,013 (92.01%) | 36,738,733 (89.79%) | 908,280 (2.22%) | 19,477,914 (47.61%) | 19,493,257 (47.64%) |
| Leaf-1 | 44,055,806 | 38,029,067 (86.32%) | 37,139,190 (84.30%) | 889,877 (2.02%) | 19,659,204 (44.62%) | 19,670,268 (44.65%) |
| Leaf-2 | 47,831,212 | 41,891,652 (87.58%) | 40,946,098 (85.61%) | 945,554 (1.98%) | 21,643,247 (45.25%) | 21,653,434 (45.27%) |
| Leaf-3 | 42,097,252 | 39,024,975 (92.70%) | 38,160,365 (90.65%) | 864,610 (2.05%) | 20,139,544 (47.84%) | 20,135,242 (47.83%) |

Table S18 Annotation statistics of novel genes assembled from transcriptome data

| Annotated Databases | Number of Novel Gene |
| --- | --- |
| COG | 65 |
| GO | 413 |
| KEGG | 367 |
| KOG | 266 |
| Pfam | 387 |
| Swiss-Prot | 298 |
| TrEMBL | 732 |
| eggNOG | 528 |
| nr | 735 |
| All | 790 |
