# Supplementary figures and images for: A chromosome level genome, as well as transcriptomes and metabolomes, insights into genome evolution and the biosynthesis of kaempferol and kaempferol derivatives in Impatiens balsamina (Balsaminaceae)
Source: Front Plant Sci. 2026 Feb 11;17:1725789. doi: 10.3389/fpls.2026.1725789 (PMC12932442; doi:10.3389/fpls.2026.1725789)

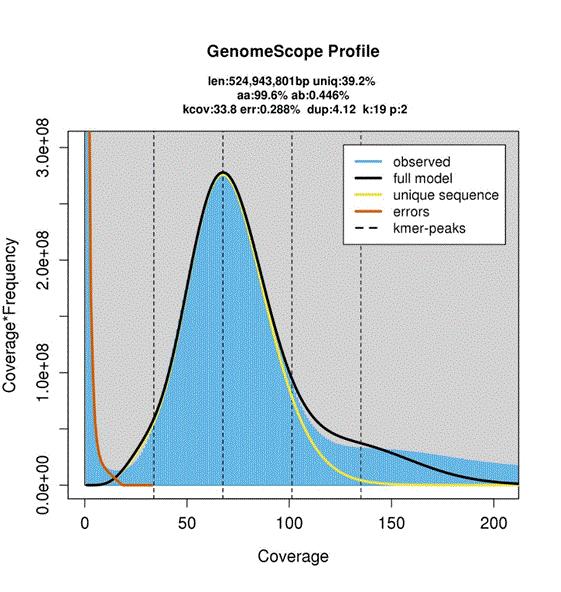

Supplement: Supplementary Data Sheet 1 — Integrated functional annotation for predicting protein-coding genes. [file DataSheet1.zip › all supplementary files/Fig. S1 K-mer analysis of the genome of I. balsamina(1).jpg]

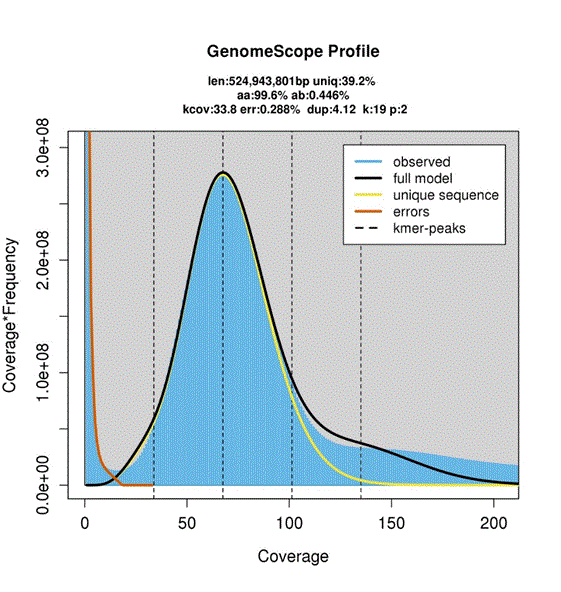

Supplement: Supplementary Data Sheet 1 — Integrated functional annotation for predicting protein-coding genes. [file DataSheet1.zip › all supplementary files/Fig. S1 K-mer analysis of the genome of I. balsamina.gif]

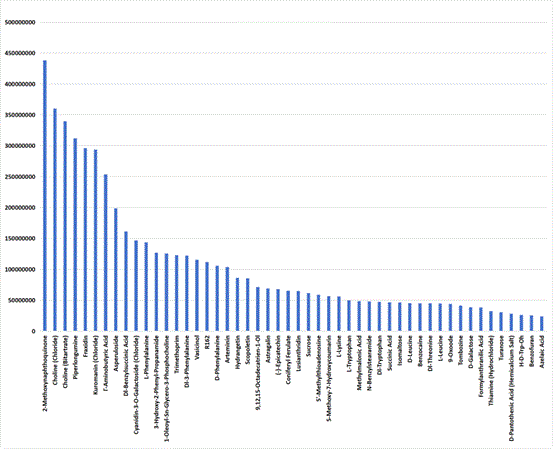

Supplement: Supplementary Data Sheet 1 — Integrated functional annotation for predicting protein-coding genes. [file DataSheet1.zip › all supplementary files/Fig. S10 Top 50 metabolites in terms of content in roots.gif]

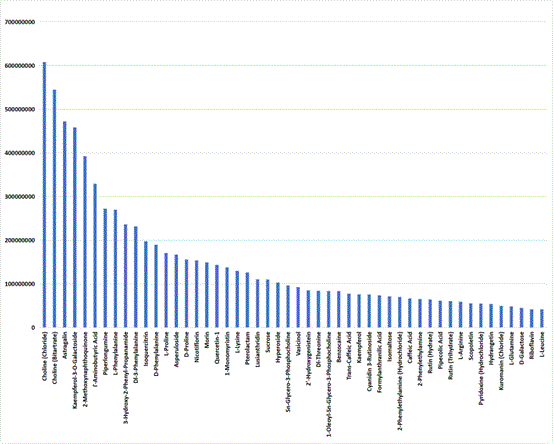

Supplement: Supplementary Data Sheet 1 — Integrated functional annotation for predicting protein-coding genes. [file DataSheet1.zip › all supplementary files/Fig. S11 Top 50 metabolites in terms of content in leaves.gif]

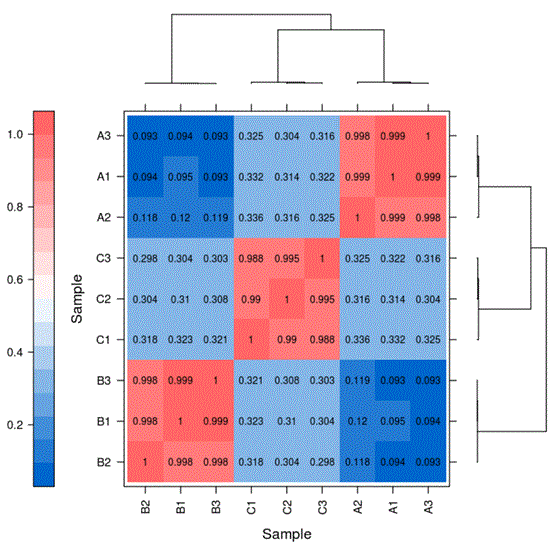

Supplement: Supplementary Data Sheet 1 — Integrated functional annotation for predicting protein-coding genes. [file DataSheet1.zip › all supplementary files/Fig. S12 Spearman rank correlation analysis of the transcript profiles of the roots, leaves, and flowers of I. balsamina.gif]

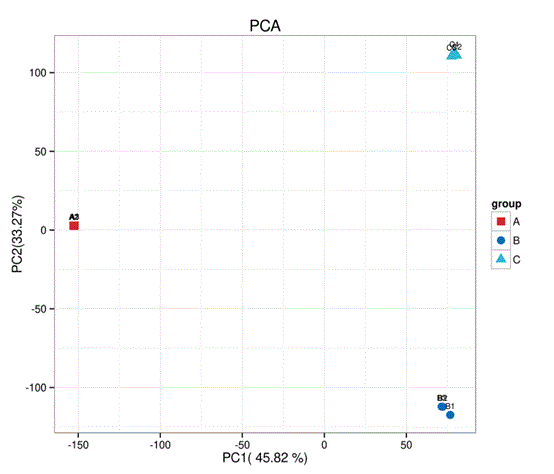

Supplement: Supplementary Data Sheet 1 — Integrated functional annotation for predicting protein-coding genes. [file DataSheet1.zip › all supplementary files/Fig. S13 Principal component analysis of the transcript profiles of the roots, leaves, and flowers of I. balsamina.gif]

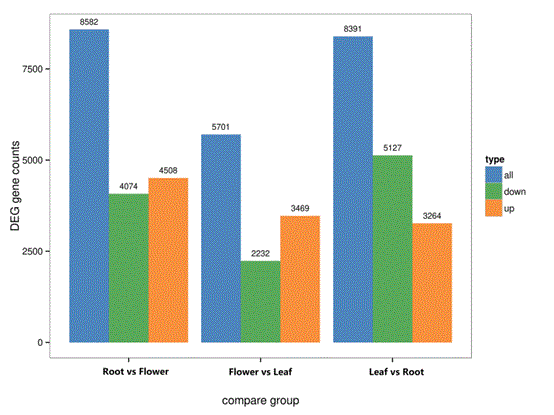

Supplement: Supplementary Data Sheet 1 — Integrated functional annotation for predicting protein-coding genes. [file DataSheet1.zip › all supplementary files/Fig. S14 Differentially expressed genes in the comparison among the roots, leaves, and flowers of I. balsamina.gif]

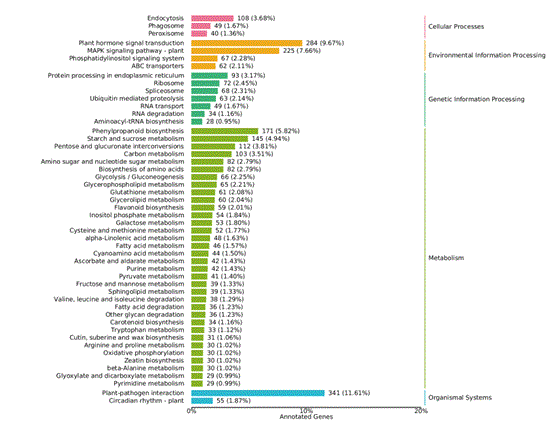

Supplement: Supplementary Data Sheet 1 — Integrated functional annotation for predicting protein-coding genes. [file DataSheet1.zip › all supplementary files/Fig. S15 KEGG pathway enrichment analysis of differentially expressed genes in the comparison between roots and flowers.gif]

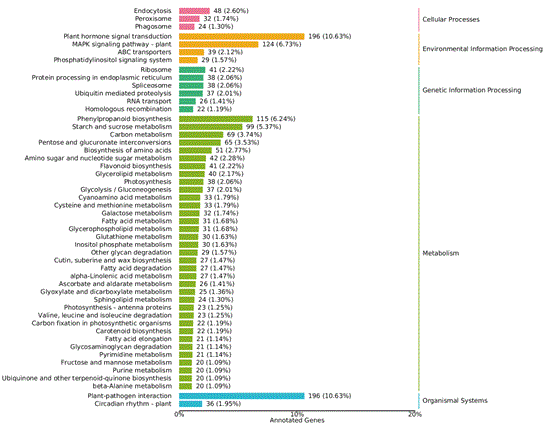

Supplement: Supplementary Data Sheet 1 — Integrated functional annotation for predicting protein-coding genes. [file DataSheet1.zip › all supplementary files/Fig. S16 KEGG pathway enrichment analysis of differentially expressed genes in the comparison between flowers and leaves.gif]

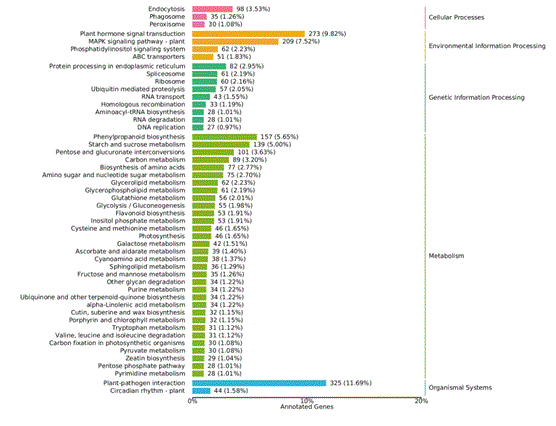

Supplement: Supplementary Data Sheet 1 — Integrated functional annotation for predicting protein-coding genes. [file DataSheet1.zip › all supplementary files/Fig. S17 KEGG pathway enrichment analysis of differentially expressed genes in the comparison between leaves and roots.gif]

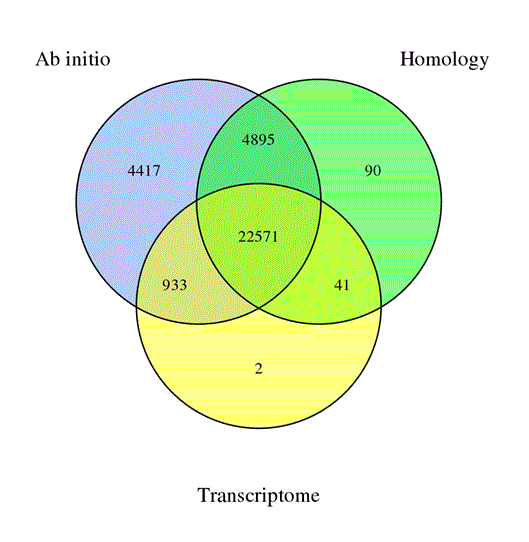

Supplement: Supplementary Data Sheet 1 — Integrated functional annotation for predicting protein-coding genes. [file DataSheet1.zip › all supplementary files/Fig. S2 Venn diagram for predicting protein-coding genes.gif]

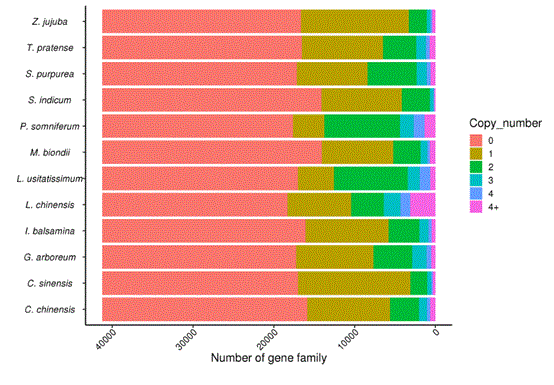

Supplement: Supplementary Data Sheet 1 — Integrated functional annotation for predicting protein-coding genes. [file DataSheet1.zip › all supplementary files/Fig. S3 Numerical statistics for each gene family in each species.gif]

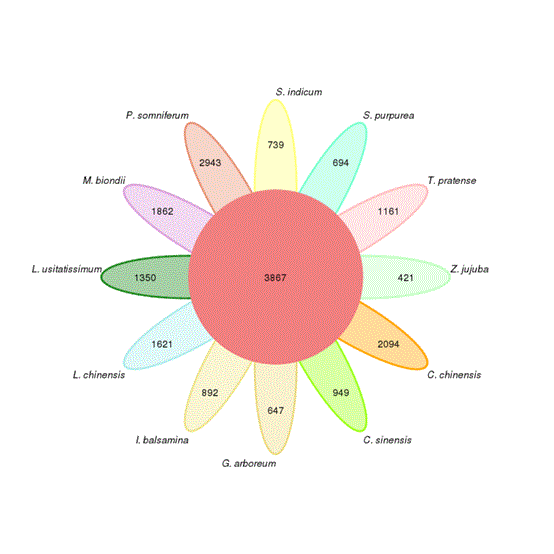

Supplement: Supplementary Data Sheet 1 — Integrated functional annotation for predicting protein-coding genes. [file DataSheet1.zip › all supplementary files/Fig. S4 Drawing of the gene families of each species.gif]

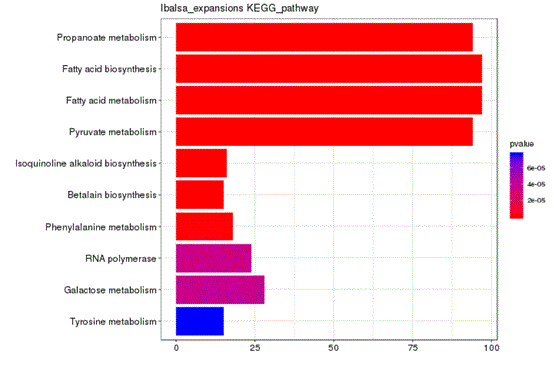

Supplement: Supplementary Data Sheet 1 — Integrated functional annotation for predicting protein-coding genes. [file DataSheet1.zip › all supplementary files/Fig. S5 KEGG enrichment of expanded gene families of I. balsamina.gif]

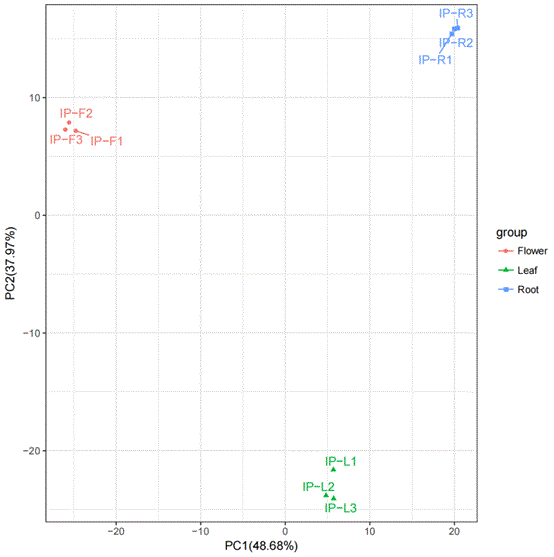

Supplement: Supplementary Data Sheet 1 — Integrated functional annotation for predicting protein-coding genes. [file DataSheet1.zip › all supplementary files/Fig. S6 Principal component analysis of the metabolite profiles of the roots, leaves, and flowers of I. balsamina.gif]

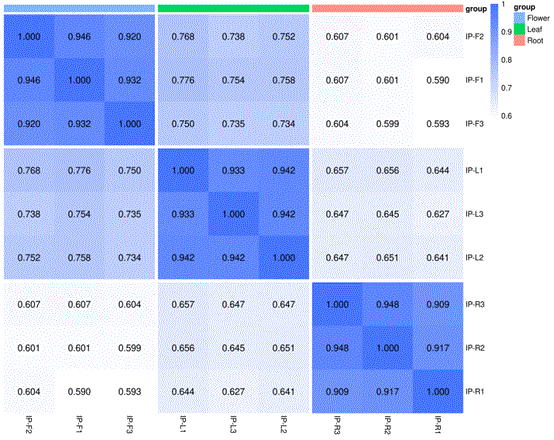

Supplement: Supplementary Data Sheet 1 — Integrated functional annotation for predicting protein-coding genes. [file DataSheet1.zip › all supplementary files/Fig. S7 Spearman rank correlation analysis of the metabolite profiles of the roots, leaves, and flowers of I. balsamina.gif]

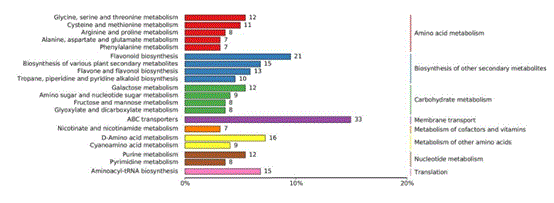

Supplement: Supplementary Data Sheet 1 — Integrated functional annotation for predicting protein-coding genes. [file DataSheet1.zip › all supplementary files/Fig. S8 Top 20 KEGG-annotated metabolic pathways.gif]

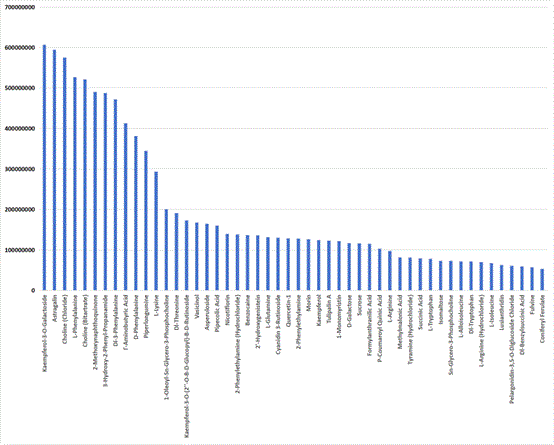

Supplement: Supplementary Data Sheet 1 — Integrated functional annotation for predicting protein-coding genes. [file DataSheet1.zip › all supplementary files/Fig. S9 Top 50 metabolites in terms of content in flowers.gif]
